# Drug Pair of Astragali Radix–Ligustri Lucidi Fructus Alleviates Acute Kidney Injury in Mice Induced by Ischemia–Reperfusion Through Inhibiting Ferroptosis

**DOI:** 10.3390/ph18060789

**Published:** 2025-05-25

**Authors:** Xuanhe Liu, Dan Zhang, Yuting Xie, Mengdan Wang, Xiaochun Chen, Weijie Yu, Yuming Ma, Jia Zeng, Qixuan Long, Guangrui Huang, Jie Geng, Anlong Xu

**Affiliations:** 1 School of Life Sciences, Beijing University of Chinese Medicine, Beijing 100029, China; liuxuanhe1998@163.com (X.L.); zd17801127553@163.com (D.Z.); nanchuqi@163.com (M.W.); cxc13173237782@163.com (X.C.); zengjia09@163.com (J.Z.); hgr@bucm.edu.cn (G.H.); 2 School of Chinese Pharmacy, Beijing University of Chinese Medicine, Beijing 100029, China; 18398214650@163.com (Y.X.); mayuming0416@163.com (Y.M.); 17200230804@163.com (Q.L.); 3 School of Traditional Chinese Medicine, Beijing University of Chinese Medicine, Beijing 100029, China; ywj261423340@163.com

**Keywords:** acute kidney injury (AKI), ischemia–reperfusion injury (IRI), ferroptosis inhibition, glutathione peroxidase 4 (GPX4), mitochondrial protection, network pharmacology, molecular docking

## Abstract

**Background:** Acute kidney injury (AKI), characterized by high morbidity and mortality, is primarily caused by renal ischemia–reperfusion injury (RIRI). Ferroptosis plays a key role in RIRI, yet its underlying mechanisms remain unclear. The drug pair of Astragali Radix–Ligustri Lucidi Fructus (DAL) shows promise in renal diseases, but its protective effects against RIRI and associated molecular pathways via ferroptosis inhibition are unknown. This study aimed to investigate DAL’s therapeutic effects on RIRI and its mechanisms. **Methods**: A mouse model of bilateral renal ischemia–reperfusion was established. Renal function (serum creatinine, Scr; blood urea nitrogen, BUN), inflammatory cytokines (TNF-α, IFN-γ, IL-6), ferroptosis markers (GPX4, MDA, GSH, tissue iron), and pathological damage were evaluated. Transcriptomic sequencing and electron microscopy analyzed gene pathways and mitochondrial structure. In HK-2 cells, oxygen–glucose deprivation/reoxygenation (OGD/R) and RSL3-induced ferroptosis models were used to assess DAL-containing serum effects via cell viability, GPX4 expression, and mitochondrial morphology. LC-MS analyzed DAL’s chemical components, and network pharmacology predicted ferroptosis-related targets. **Results**: DAL significantly reduced Scr/BUN levels, alleviated tubular injury, fibrosis, and apoptosis, and downregulated inflammatory cytokines and damage markers. It inhibited ferroptosis by upregulating GPX4, decreasing MDA/tissue iron, and increasing GSH. Transcriptomics revealed enrichment in lipid metabolism pathways. DAL restored the mitochondrial cristae structure; DAL-containing serum improved cell viability, blocked RSL3-induced GPX4 downregulation, and mitigated mitochondrial dysfunction. Network pharmacology identified DAL’s potential active components and targets. Molecular docking validated binding affinity and interaction patterns of active components with targets. **Conclusions**: DAL protects against RIRI by upregulating GPX4, preserving the mitochondrial structure, and inhibiting ferroptosis, highlighting its therapeutic potential for AKI prevention and treatment.

## 1. Introduction

Ischemia–reperfusion injury (IRI), a pathological process prevalent in surgical procedures and disease treatments involving multiple organs such as the heart, brain, and kidneys, leads to tissue cell damage, organ dysfunction, and severely impacts patient recovery and quality of life [1]. Renal ischemia–reperfusion injury (RIRI) is a primary cause of acute kidney injury (AKI). According to the KDIGO (Kidney Disease: Improving Global Outcomes) guidelines, AKI is defined as a serum creatinine increase of ≥0.3 mg/dL within 48 h, a 1.5-fold elevation from baseline within 7 days, or a sustained urine output <0.5 mL/kg/h for 6 h [2,3]. With a high global incidence and mortality—affecting approximately 13.3 million patients annually [4]—AKI has emerged as a critical public health challenge. Current treatments, including supportive care, etiological therapy, continuous renal replacement therapy (CRRT), and intermittent hemodialysis (IHD) [5,6], have failed to significantly reduce mortality, underscoring the urgent need for innovative therapeutic approaches. Recent studies highlight a pivotal role of ferroptosis in IRI pathogenesis [7,8].

Ferroptosis, a newly identified iron-dependent atypical form of programmed cell death [9], distinct from traditional pathways like apoptosis and necrosis, is characterized by iron-overload-induced lipid peroxidation cascades [10]. Mechanistically, the abnormal accumulation of intracellular Fe^2+^, catalyzing Fenton reactions, drives lipid peroxidation of polyunsaturated fatty acids in cell membranes, generating toxic products, such as 4-hydroxynonenal, that disrupt membrane integrity. The depletion of glutathione (GSH) and inhibition of glutathione peroxidase 4 (GPX4), key components of the antioxidant system, render cells unable to clear lipid peroxides, constituting a critical mechanism of ferroptosis [11]. Morphologically, it is marked by mitochondrial shrinkage, increased membrane density, reduced or absent cristae, and outer membrane rupture [12]. Induced by iron overload, antioxidant deficiency, or drugs targeting GPX4 or system Xc^−^, and inhibited by iron chelators, antioxidants, or GPX4 overexpression, ferroptosis participates in multiple pathological processes, including cancer, neurodegenerative diseases, and IRI, making its characteristic mechanisms potential therapeutic targets [13].

In RIRI, iron metabolic imbalance in renal tubular epithelial cells leads to excessive reactive oxygen species (ROS) accumulation and GPX4 inactivation, triggering membrane phospholipid peroxidation and subsequent ferroptotic cell death [14,15,16]. Elucidating the correlation between ferroptosis and IRI is crucial for understanding the pathogenesis of AKI. Notably, crosstalk between ferroptotic regulatory networks and classical apoptotic pathways suggests its potential as a dual target for AKI therapy [17,18]. Deciphering the molecular mechanisms of ferroptosis in IRI not only deepens our understanding of AKI pathology but also provides a theoretical basis for developing novel interventions targeting iron metabolism.

Traditional Chinese medicine (TCM), with its unique theoretical and therapeutic frameworks, has increasingly demonstrated advantages in AKI treatment [19,20]. The herbal pair Astragali Radix and Ligustri Lucidi Fructus, the core components of Zhenqi Fuzheng Granules, exhibits immunomodulatory and antitumor activities [21,22] and has been used in treating renal diseases [23,24] and ulcerative colitis [25]. Individual components of this formula have shown therapeutic effects against ischemia–reperfusion injury: astragaloside IV, a major active component of Astragali Radix, mitigates renal IRI via interactions between HIF-1α and the NF-κB (p65)/Smad 7 pathway [26]; salidroside from Ligustri Lucidi Fructus alleviates lung IRI via the JAK2/STAT3 signaling pathway [27]. Notably, these two herbs show potential for synergistic effects in combating IRI. However, the role of the drug pair of Astragali Radix–Ligustri Lucidi Fructus (DAL) in renal IRI and its molecular mechanisms—particularly whether it exerts protective effects by regulating ferroptosis—remain uninvestigated.

This study systematically analyzes the pathological characteristics of IRI and AKI, aiming to explore the therapeutic mechanisms of DAL. By focusing on the association between ferroptosis and renal injury, we investigate whether DAL mitigates RIRI by inhibiting ferroptosis in renal tubular epithelial cells. Our findings aim to provide a theoretical foundation for the clinical application of DAL in ischemic renal injury.

## 2. Results

### 2.1. DAL Ameliorates I/R-Induced AKI in Mice

**Figure 1:**
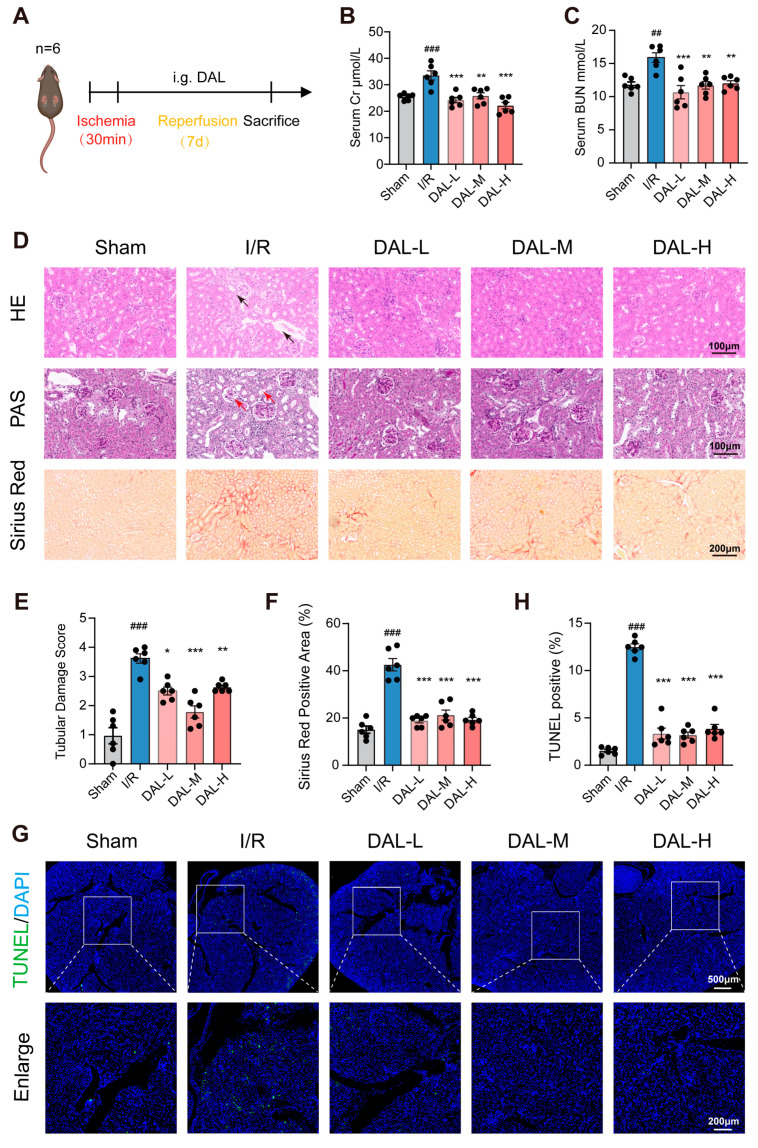
DAL alleviates post-ischemia/reperfusion renal injury. (**A**) Schematics of the I/R experimental protocol (*n* = 6); (**B**) Scr concentration; (**C**) BUN concentration; (**D**) representative micrographs of H&E, PAS, and Sirius Red staining; scale bars, 100 μm/200 μm; (**E**) quantification of tubular injury scores; (**F**) quantification of Sirius Red-positive area percentage; (**G**) representative fluorescent images of TUNEL staining; scale bars, 500 μm/200 μm; (**H**) quantification of TUNEL-positive area percentage; I/R vs. Sham: ## *p* < 0.01, ### *p* < 0.001; DAL vs. I/R: * *p* < 0.05, ** *p* < 0.01, *** *p* < 0.001; data are presented as mean ± SEM.

To evaluate the therapeutic effects of DAL on acute kidney injury, bilateral renal ischemia–reperfusion (I/R) surgery was performed in C57 mice, followed by daily intragastric administration of DAL for 7 days. Mice were euthanized, and serum and kidney tissues were collected for analysis (Figure 1A). Serum creatinine (Scr) and blood urea nitrogen (BUN), widely used as indicators of acute kidney injury [28,29], were significantly reduced in the low-, medium-, and high-dose DAL groups compared to the model group (Figure 1B,C). Pathological sections stained with H&E and PAS revealed that the model group exhibited renal injury phenotypes, including renal tubular brush border loss, luminal dilation, vacuolization (red arrows), and glomerular atrophy (black arrows), which were alleviated following DAL treatment (Figure 1D,E). Sirius Red staining showed that DAL reduced I/R-induced renal fibrosis (Figure 1F). TUNEL staining indicated that DAL significantly attenuated renal cell death caused by I/R injury (Figure 1G,H).

**Figure 2:**
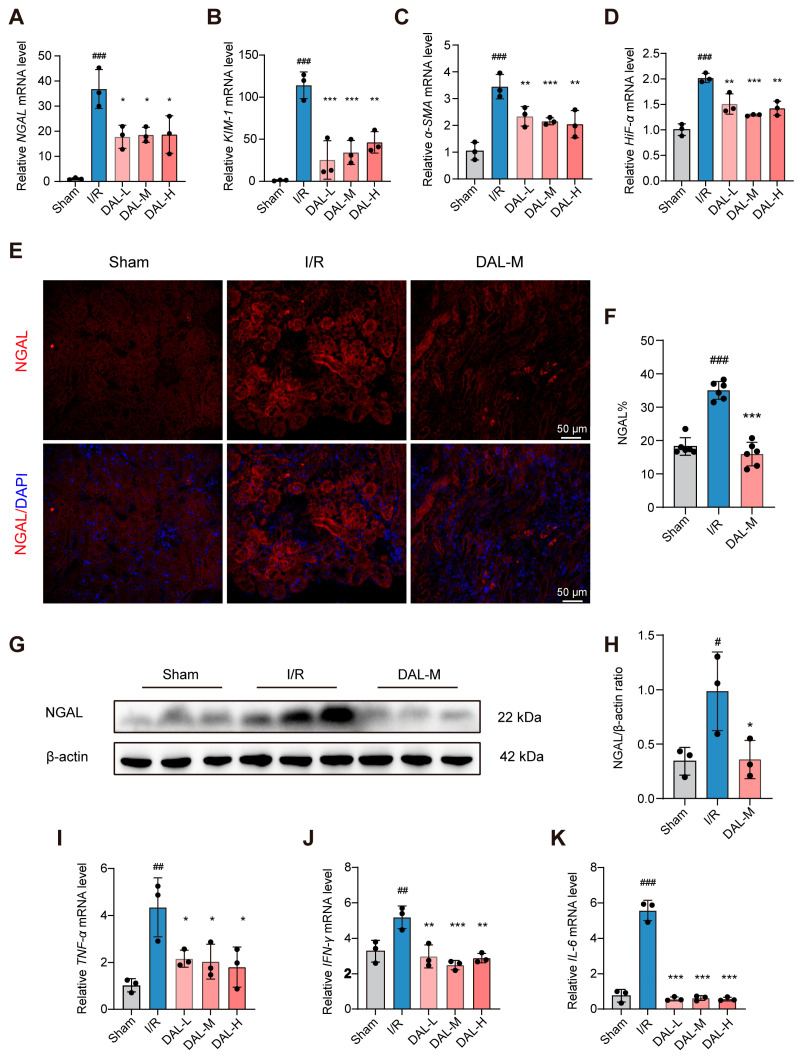
DAL alleviates I/R-induced AKI in mice; (**A**–**D**) mRNA levels of NGAL, KIM-1, HIF-1α, and α-SMA; (**E**) representative immunofluorescence images of NGAL; scale bars, 50 μm; (**F**) quantification of NGAL-positive area percentage; (**G**,**H**) Western blot results showing NGAL expression levels; (**I**–**K**) mRNA levels of TNF-α, IFN-γ, and IL-6; I/R vs. Sham: # *p*< 0.05, ## *p* < 0.01, ### *p* < 0.001; DAL vs. I/R: * *p* < 0.05, ** *p* < 0.01, *** *p* < 0.001; data are presented as mean ± SEM.

RT-qPCR results showed that tubular injury markers NGAL and KIM-1 [30], hypoxia-inducible factor HIF-1α, and fibrosis marker α-SMA were significantly upregulated after surgery (Figure 2A–D). These alterations were reversed by DAL treatment, demonstrating its significant efficacy against I/R-induced pathological processes. Notably, the therapeutic effects of DAL were not dose-dependent, and the medium-dose DAL group (DAL-M, equivalent to the human therapeutic dosage) was selected for further studies. Western blotting (WB) and immunofluorescence (IF) analyses confirmed that DAL downregulated NGAL expression (Figure 2E–H). Additionally, DAL significantly reduced the mRNA levels of proinflammatory cytokines TNF-α, IFN-γ, and IL-6 in renal tissues (Figure 2I–K).

Additionally, to evaluate the long-term effects of DAL, continuous administration for 14 days post-surgery was performed (Supplementary Figure S1A). Notably, Scr and BUN levels in the DAL-treated groups were significantly reduced compared to the model group (Supplementary Figure S1B,C), consistent with previous reports [29]. However, histological staining (H&E, PAS) revealed persistent renal injury phenotypes in the model group (Supplementary Figure S1D–F), while long-term DAL administration still demonstrated the amelioration of I/R-induced damage, indicating the sustained therapeutic effects of DAL on chronic renal injury.

### 2.2. DAL Alleviates I/R-Induced AKI in Mice by Inhibiting Ferroptosis

**Figure 3:**
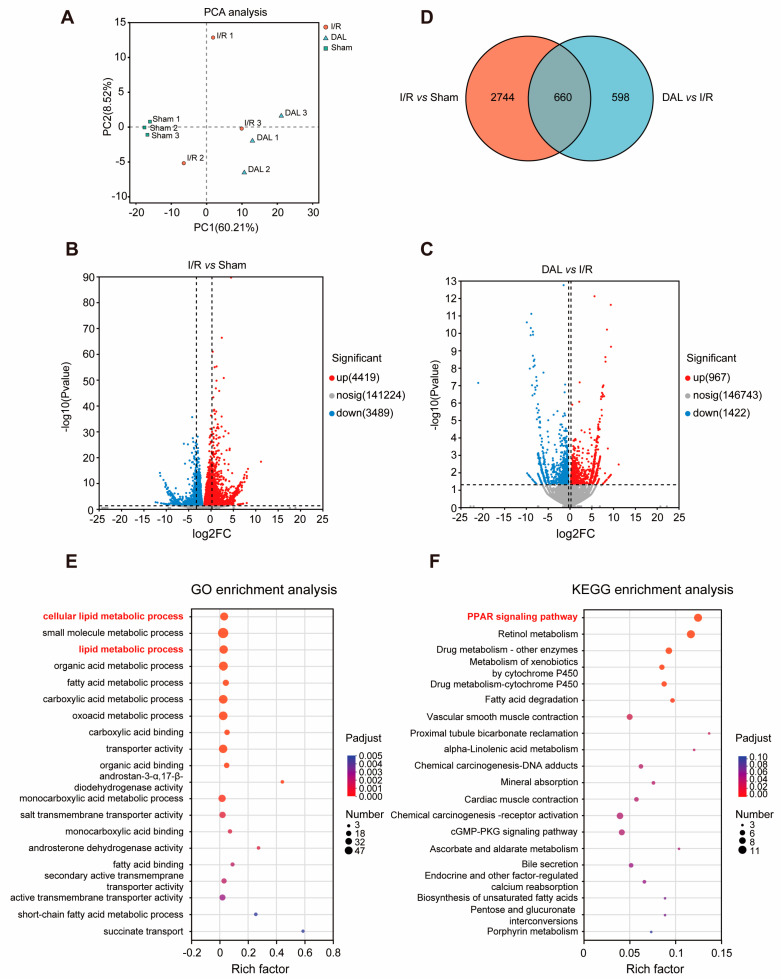
Transcriptome sequencing results of Sham, I/R, and DAL. (**A**) PCA plot of sample gene expression abundance; (**B**,**C**) volcano plots of gene expression abundance across samples; (**D**) Venn diagram of differentially expressed genes; (**E**) top 20 biological functions from GO functional enrichment analysis of target genes; (**F**) top 20 biological functions from KEGG pathway enrichment analysis of target genes.

Accumulating evidence in recent years has linked renal I/R injury in mice to ferroptosis, with ferroptosis inhibition shown to ameliorate I/R damage. To explore this, we performed RNA sequencing (RNA-seq) on mouse kidneys after I/R. Principal component analysis (PCA) of the samples (Figure 3A) revealed that most samples exhibited low dispersion and concentrated distribution, indicating minimal technical variation. Differentially expressed genes (DEGs) were identified using DESeq2 software (1.48.1) with thresholds set as adjusted *p*-value (padj) < 0.05 and |log2 fold change (FC)| ≥ 1.2. Volcano plots visualized DEGs meeting the criteria of *p* < 0.05 and |log2FC| > 1.2 (Figure 3B,C), and intersection analysis yielded 660 DEGs (Figure 3D). GO pathway enrichment analysis showed the significant enrichment of DEGs in cellular lipid metabolic processes (Figure 3E), while KEGG analysis highlighted involvement in the PPAR signaling pathway (Figure 3F). Given that PPAR family members regulate fatty acid oxidation, cholesterol metabolism, inflammation, and cell differentiation—playing a critical role in lipid homeostasis [31]—and that lipid peroxidation is a direct trigger of ferroptosis [11], we hypothesize that DAL alleviates I/R-induced AKI in mice by inhibiting ferroptosis.

**Figure 4:**
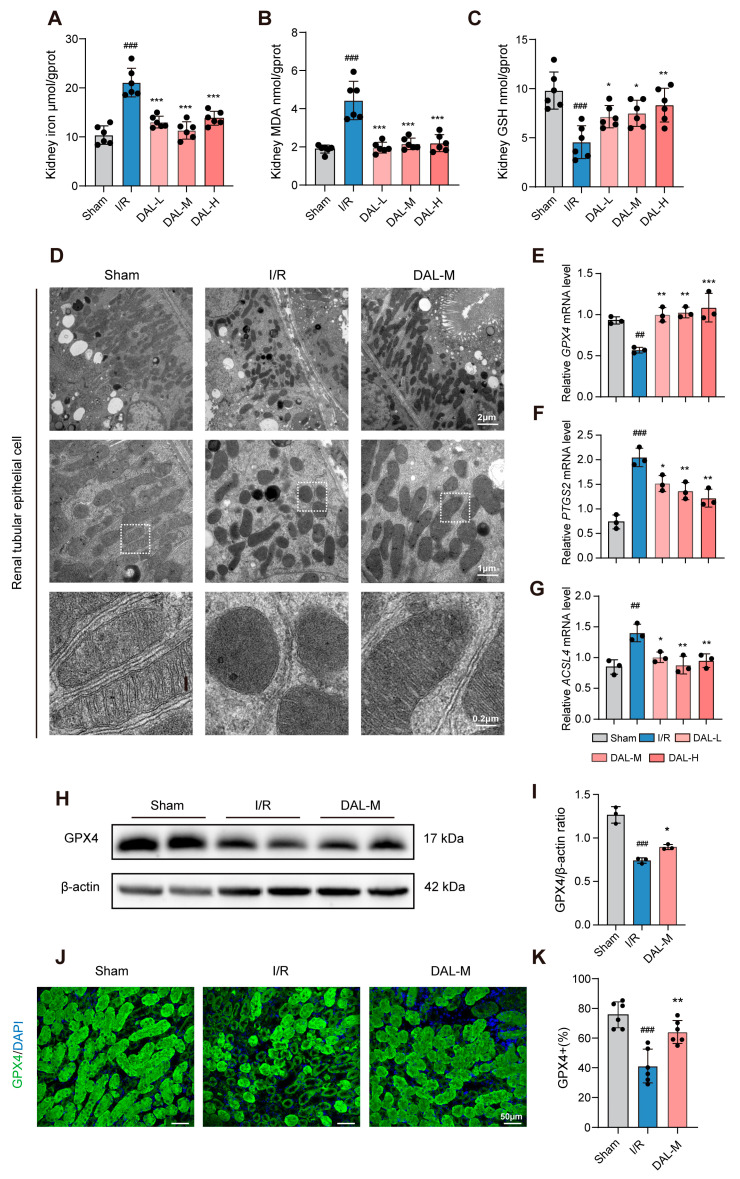
DAL alleviates I/R-induced AKI in mice. (**A**–**C**) Levels of tissue iron, MDA, and GSH in mouse kidneys; (**D**) representative images of renal cortical mitochondria captured by transmission electron microscopy; scale bars, 2 μm/1 μm/0.2 μm; (**E**–**G**) mRNA levels of GPX4, ACSL4, and PTGS2; (**H**,**I**) Western blot results showing GPX4 expression levels and quantitative analysis; (**J**) representative immunofluorescence micrographs of GPX4; scale bars, 50 μm; (**K**) quantification of GPX4-positive area percentage; I/R vs. Sham: ## *p* < 0.01, ### *p* < 0.001; DAL, Fer-1 vs. I/R: * *p* < 0.05, ** *p* < 0.01, *** *p* < 0.001; Data are presented as mean ± SEM.

To validate the transcriptomic findings, we measured ferroptosis-related indices in renal tissues. Compared to the I/R group, DAL reduced tissue iron and malondialdehyde (MDA) levels while increasing glutathione (GSH) levels (Figure 4A–C), indicating decreased ferroptotic risk in renal cells. Ferroptosis is associated with mitochondrial morphological and functional alterations [32], such as shrinkage, rounding, cristae loss, and membrane rupture [33]. Transmission electron microscopy of renal cortical mitochondria (Figure 4D) showed extensive damage in the I/R group—including cristae loss and membrane rupture with matrix release—whereas DAL-treated samples exhibited increased mitochondria with preserved cristae, demonstrating protective effects on mitochondrial structure.

RT-qPCR analysis of key ferroptosis-related molecules (GPX4, ACSL4, PTGS2) revealed that DAL restored their expression levels (Figure 4E–G). Given GPX4’s pivotal role in ferroptosis, Western blotting and immunofluorescence confirmed that DAL upregulated GPX4 protein expression after I/R (Figure 4H–K). Following long-term administration, ferroptosis-related indices and GPX4 expression normalized, except for a persistent reduction in NGAL (Supplementary Figure S1G–L).

Subsequently, the ferroptosis inhibitor (Fer-1) was employed as a positive control [34,35] to compare the therapeutic effects of DAL and Fer-1 in I/R mice (Supplementary Figure S2A). Analysis of serum markers, renal histology, and ferroptosis-related proteins/mRNA (Supplementary Figure S2B–H) corroborated DAL’s efficacy in alleviating I/R injury and inhibiting ferroptosis. H&E staining of liver tissues showed no hepatotoxicity from DAL (Supplementary Figure S2E), confirming its safety.

DAL-M and Fer-1 exhibited comparable effects on ferroptosis-related indices (Supplementary Figure S3A–C), with both upregulating GPX4 and downregulating NGAL (Supplementary Figure S3D–H). Electron microscopy further showed that both interventions preserved the mitochondrial structure. Collectively, these results support the hypothesis that DAL inhibits post-I/R ferroptosis by upregulating GPX4.

### 2.3. DAL-Containing Serum Improves Cell Viability and Attenuates Ferroptosis After OGD/R

**Figure 5:**
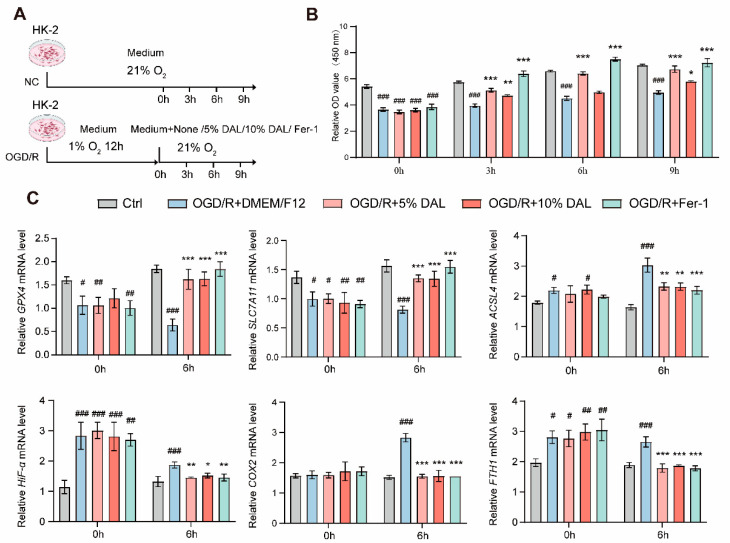
The DAL-containing serum can restore the viability of OGD/R-induced HK-2 cells and alleviate ferroptosis. (**A**) Schematic of the OGD/R experimental protocol in HK-2 cells; (**B**) measurement of relative cell viability after OGD/R; (**C**) relative mRNA expression levels of GPX4, SLC7A11, ACSL4, FTH1, HIF-1α, and COX2 in cells at 0 h and 6 h post-reoxygenation; RSL3 vs. NC: # *p*< 0.05, ## *p* < 0.01, ### *p* < 0.001; DAL, Fer-1 vs. RSL3: * *p* < 0.05, ** *p* < 0.01, *** *p* < 0.001; data are presented as mean ± SEM.

To validate the effects of DAL-derived blood components, an oxygen–glucose deprivation and reoxygenation (OGD/R) model was established in HK-2 renal tubular epithelial cells [36] (Figure 5A). Hypoxia-induced HK-2 cells were treated with DAL-containing serum at concentrations of 5% and 10%, or with Fer-1-containing complete medium. Cell viability assays showed that OGD/R significantly reduced cell viability, while the DAL-containing serum intervention group exhibited notable cytoprotective effects, with superior repair-promoting efficacy compared to the control group. However, this enhancement became less significant over extended culture periods (Figure 5B).

Meanwhile, mRNA analysis of HK-2 cells collected immediately after reoxygenation and at 6 h revealed typical ferroptotic characteristics following OGD/R: downregulation of antioxidant system key genes (GPX4, SLC7A11) and upregulation of lipid peroxidation-related genes (ACSL4, FTH1). Hypoxia marker HIF-1α was significantly elevated during the ischemic phase, while inflammatory mediator COX2 was specifically activated after reoxygenation (Figure 5C). DAL-containing serum reversed these abnormal expression patterns, suggesting that DAL inhibits ferroptosis and inflammatory cascades through multi-target synergistic effects. The 5% concentration group showed optimal regulatory effects, likely due to the potential toxicity of certain components at high concentrations or at target saturation.

### 2.4. DAL-Containing Serum Reverses RSL3-Induced Reduction in GPX4 Levels and Improves Mitochondrial Function

**Figure 6:**
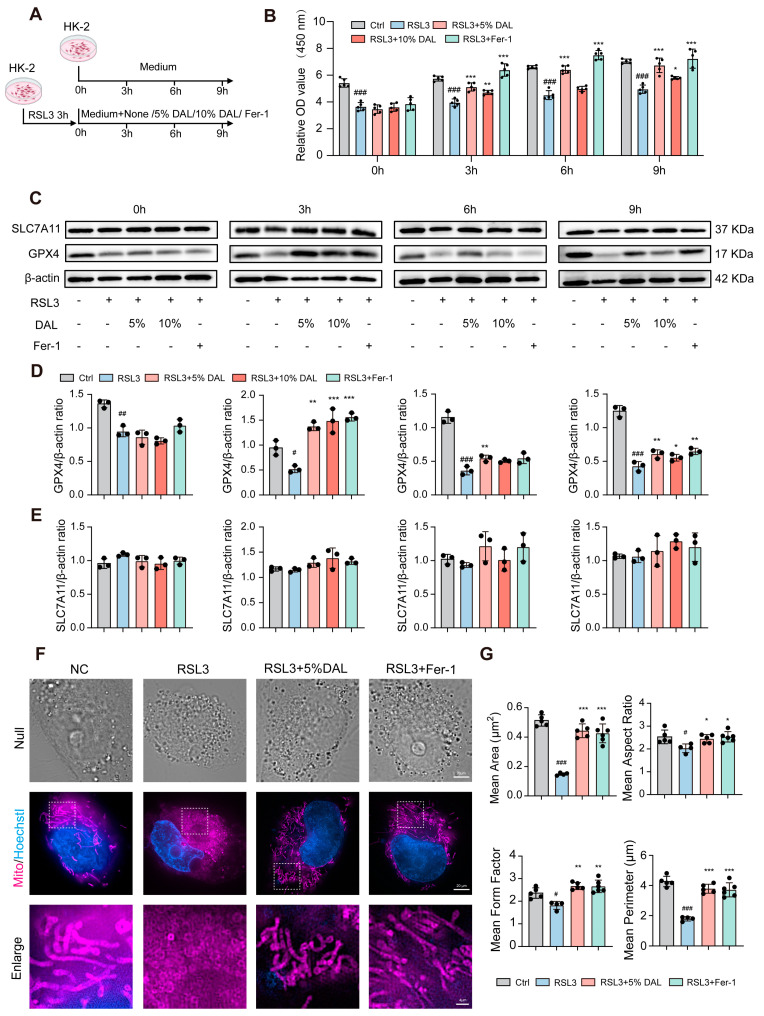
The medicated serum of DAL can reverse the decrease in the content of GPX4 in HK-2 cells induced by RSL3 and improve mitochondrial function. (**A**) Schematic of the HK-2 ferroptosis experimental protocol; (**B**) measurement of HK-2 cell viability; (**C**–**E**) Western blot expression levels of GPX4 and SLC7A11; (**F**) representative images of mitochondrial fluorescence staining; scale bars, 20 μm/4 μm; (**G**) statistical analysis of mitochondrial morphological parameters; OGD/R vs. NC: # *p* < 0.05, ## *p* < 0.01, ### *p* < 0.001; DAL, Fer-1 vs. OGD/R: * *p* < 0.05, ** *p* < 0.01, *** *p* < 0.001; data are presented as mean ± SEM.

To further validate the direct regulatory effects of DAL-containing serum on ferroptosis, an in vitro model was established using the classic ferroptosis inducer RSL3, a direct inhibitor of GPX4 that induces lipid peroxidation cascades by covalently binding to GPX4 active sites [10]. HK-2 cells were treated with RSL3 for 3 h, followed by intervention with DAL-containing serum or Fer-1 (Figure 6A). Experimental results showed that RSL3 treatment significantly reduced cell viability, which recovered over time following intervention with DAL-containing serum or Fer-1. Similarly to the OGD/R model, the 5% concentration group exhibited the best recovery effect (Figure 6B). Western blotting revealed a significant decrease in GPX4 levels after RSL3 treatment, which recovered following DAL-containing serum treatment, indicating that DAL enhances cellular resistance to ferroptosis by upregulating GPX4. The expression of SLC7A11, another key ferroptosis-related protein (cysteine–glutamate transporter), was unaffected by RSL3 or DAL (Figure 6C–E).

Mitochondrial function assays showed that RSL3 treatment induced significant morphological changes, including a characteristic shortening and rounding of the mitochondria. In contrast, the DAL-containing serum coincubation group exhibited a significantly enhanced mitochondrial fluorescence signal intensity (Figure 6F,G), suggesting the maintenance of mitochondrial membrane integrity. This finding is consistent with the in vivo observations of the restored mitochondrial cristae structure in mouse kidneys via transmission electron microscopy, further validating that DAL inhibits ferroptosis by protecting mitochondrial function.

### 2.5. Deciphering the Material Basis of DAL’s Anti-Ischemia-Reperfusion Injury Effects via Ferroptosis Inhibition

To identify specific components in the DAL that alleviate I/R injury via ferroptosis inhibition, we selected astragaloside IV (AS-IV from Astragali Radix) and salidroside (SAL from Ligustri Lucidi Fructus) based on previous reports [37] and studies on I/R injury mitigation. Animal and cell experiments showed that AS-IV and SAL alleviated renal injury caused by I/R (Supplementary Figure S4A–H), partially restoring tissue iron, MDA, and (GSH) levels (Supplementary Figure S4I–K). While they reduced NGAL expression, they failed to significantly restore GPX4 downregulation (Supplementary Figure S5A–C), with their overall efficacy being less pronounced than DAL. Mitochondrial fluorescence staining in cells further indicated that AS-IV and SAL had minimal protective effects against RSL3-induced mitochondrial damage during ferroptosis (Supplementary Figure S5D–H). Histological analysis of liver tissues via H&E staining confirmed no hepatotoxicity from DAL (Supplementary Figure S5E), suggesting that AS-IV and SAL may exert renoprotective effects through non-ferroptotic pathways (e.g., antioxidation/inflammation) or non-GPX4-dependent ferroptosis regulation.

**Figure 7:**
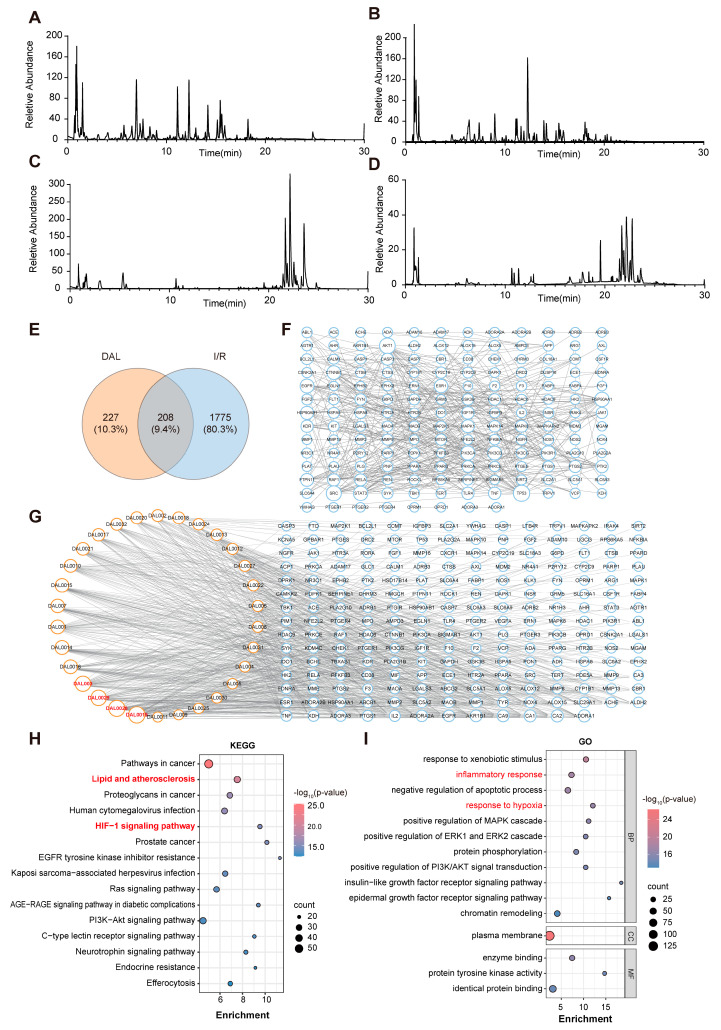
Identification of DAL and its blood-absorbed components, and network pharmacology analysis. (**A**,**B**) Base peak intensity (BPI) maps of DAL in positive (**A**)/negative (**B**) ion modes; (**C**,**D**) BPI maps of blood-entering components in positive (**C**)/negative (**D**) ion modes; (**E**) Venn diagram of DAL and I/R targets; (**F**) protein–protein interaction (PPI) network diagram of DAL component targets; (**G**) PPI network diagram of DAL components and ferroptosis-related pathways; (**H**) top 15 biological functions from KEGG pathway enrichment analysis of target genes; (**I**) top 15 biological functions from GO functional enrichment analysis of target genes.

To investigate the material basis of DAL’s ferroptosis-inhibiting effects, ultra-high-performance liquid chromatography–quadrupole orbitrap high-resolution mass spectrometry (UHPLC-Q-Orbitrap HRMS) was used to analyze DAL and its blood-enterable components. Base peak chromatograms were obtained via positive/negative ion full-scan mode (Figure 7A–D), and components were identified by matching retention times, accurate mass-to-charge ratios, and tandem mass spectrometry data (Supplementary Tables S1 and S2). Network pharmacology analysis revealed the potential mechanisms by which DAL intervenes in I/R, strongly linking its effects to ferroptosis regulation.

A Venn diagram (Figure 7E) showed the significant overlap between DAL and RIRI targets, highlighting multi-target synergism. Key nodes like HIF-1α, VEGFA, and TNF-α were identified in the PPI network diagram (Figure 7F). As a hub of hypoxic response, HIF-1α may regulate intracellular iron homeostasis by modulating iron metabolism-related genes (e.g., TFR1, FPN1), thereby influencing ferroptosis. KEGG enrichment analysis (Figure 7H) highlighted the HIF-1 signaling pathway, which intersects with critical ferroptosis regulators (ACSL4, GPX4): HIF-1α activation under hypoxia promotes lipid metabolic reprogramming and polyunsaturated fatty acid synthesis, exacerbating lipid peroxidation. GO analysis (Figure 7I) revealed enrichment in “oxidative stress regulation” and “oxidoreductase activity”, supporting the hypothesis that DAL enhances glutathione metabolism (e.g., restoring GPX4 activity) or inhibits lipid peroxidation cascades to counteract ferroptotic damage.

Additionally, the dense connectivity in the DAL component–ferroptosis pathway PPI network (Figure 7G) suggests that DAL acts through the multidimensional regulation of iron accumulation, antioxidant defense, and membrane lipid repair, ultimately mitigating mitochondrial dysfunction and iron-dependent cell death in renal IRI. Collectively, DAL likely exerts renoprotective effects by interacting with the HIF-1 signaling pathway and core ferroptosis networks, synergistically improving the hypoxic microenvironment, inhibiting iron overload, and reducing lipid peroxidation.

**Figure 8:**
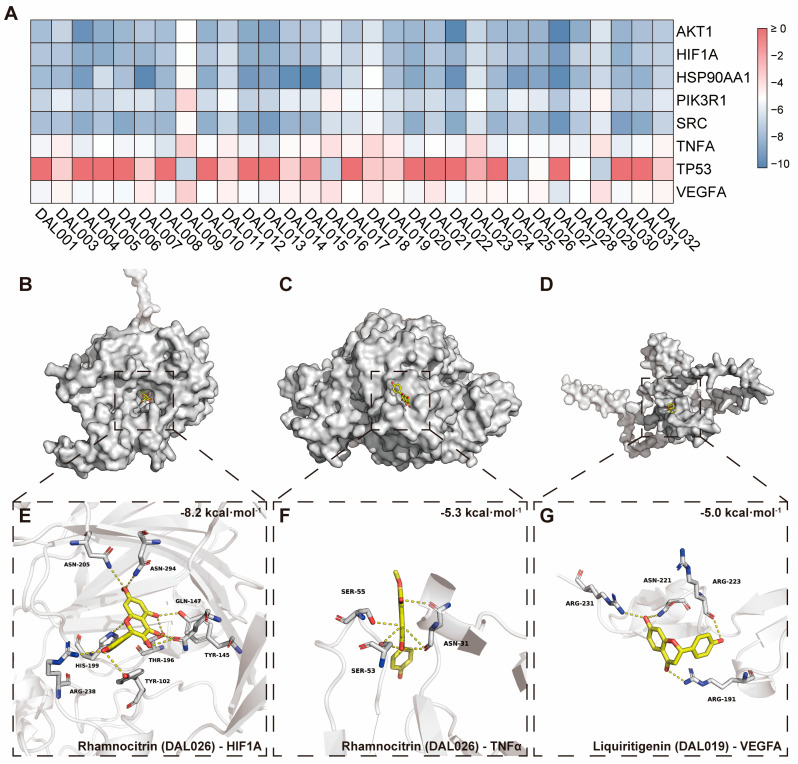
Molecular docking of active DAL components with key protein targets. (**A**) The binding energy heat map of DAL’s active components with key protein targets; (**B**–**G**) the three-dimensional binding modes between DAL’s active components and key protein targets (Proteins are displayed in gray, with active components highlighted in yellow. Oxygen atoms are colored red, nitrogen atoms in blue, and hydrogen bonds are represented by yellow dashed lines).

To further validate the relationship between active compounds of DAL and potential targets for renal ischemia–reperfusion injury (RIRI), molecular docking was performed. Integrating compound–target pathway networks, Gene Ontology (GO) enrichment analysis, protein–protein interaction (PPI) network analysis, and expression analysis of the aforementioned targets, we identified AKT1, HIF-1α, HSP90AA1, PIK3R1, SRC, TNF-α, TP53, and VEGFA as key targets mediating DAL’s therapeutic effects on RIRI. Molecular docking was conducted between DAL’s active compounds and these targets. Binding activities and probabilities were evaluated based on binding energies derived from the result files generated by the AutoDock Vina program(1.2.2). A lower binding energy between a ligand and receptor generally indicates a more stable docking conformation. Docking results showed that the binding affinities of DAL’s active compounds to these putative key targets were all less than −5 kcal·mol^−1^, indicating favorable binding capacities in RIRI treatment (Figure 8A). Compounds DAL003 (7,2’-dihydroxy-3’,4’-dimethoxyisoflavan), DAL019 (Liquiritigenin), DAL026 (Rhamnocitrin), and DAL029 (Sinapic acid), which were most frequently associated with RIRI targets, were prioritized for attention. As shown in Figure 8B–G, Rhamnocitrin exhibited particularly high binding affinities for HIF-1α and TNF-α, with values of −8.2 kcal·mol^−1^ and −5.3 kcal·mol^−1^, respectively. Liquiritigenin displayed a binding energy of −5.0 kcal·mol^−1^ with VEGFA. These findings suggest that these compounds may serve as primary components through which DAL exerts its therapeutic effects in RIRI. These structural features provided visual spatial conformation evidence for component–target interactions. Collectively, the binding energy data and three-dimensional structural predictions demonstrated that DAL26 and DAL19 exhibit potential binding activities with HIF-1α, VEGFA, and TNF-α at the molecular level, providing critical structural biological evidence for subsequent targeted mechanistic validation and pharmacodynamic studies.

## 3. Discussion

This study systematically elucidated the mechanism by which the drug pair of Astragali Radix–Ligustri Lucidi Fructus (DAL) alleviates acute kidney injury (AKI) via ferroptosis inhibition, using both animal and cell experiments. In animal studies, an I/R-induced AKI model was employed to assess Scr, BUN, inflammatory cytokines (TNF-α, IFN-γ, IL-6), ferroptosis-related indices (GPX4, MDA, GSH, tissue iron), and pathological damage. Transcriptomic sequencing analyzed differentially expressed gene pathways, while transmission electron microscopy observed the mitochondrial structure. In cell experiments, oxygen–glucose deprivation/reoxygenation (OGD/R) and RSL3-induced ferroptosis models in HK-2 cells were used to evaluate cell viability, GPX4/SLC7A11 expression, and mitochondrial morphology via fluorescent staining. UHPLC-Q-Orbitrap HRMS was applied to characterize DAL’s chemical composition, and network pharmacology predicted potential targets in ferroptosis regulation. Results showed that DAL significantly reduced Scr/BUN levels; attenuated tubular injury, fibrosis, and apoptosis; downregulated inflammatory cytokines (TNF-α, IFN-γ, IL-6) and injury markers (NGAL, KIM-1); and inhibited ferroptosis by upregulating GPX4, decreasing MDA/tissue iron, and increasing GSH. Transcriptomic analysis revealed enrichment in lipid metabolism pathways, while mitochondrial protection was demonstrated by the restored cristae structure and reversal of RSL3-induced GPX4 downregulation/mitochondrial dysfunction in HK-2 cells. Network pharmacology identified the material basis and potential targets of DAL in mitigating I/R-induced ferroptosis, providing experimental and theoretical support for its clinical application in AKI.

As a classic AKI model, the I/R model effectively mimics pathological processes in clinical scenarios like renal transplantation and shock resuscitation, serving as the primary tool for studying AKI and chronic kidney disease (CKD) [38]. Its advantage lies in the precise control of ischemia duration and reperfusion injury severity. The HK-2 cell OGD/R model complements the in vivo I/R model by recapitulating core pathological events at the cellular level—simulating energy depletion during ischemia and oxidative/mitochondrial damage during reoxygenation. While the renal I/R model evaluates renal protective effects at the organismal level, the HK-2 OGD/R model isolates cellular mechanisms, focusing on cell viability, ferroptosis-related protein expression (e.g., GPX4, SLC7A11), and mitochondrial morphology, free from systemic physiological interference. The consistency between in vivo and in vitro findings validates DAL’s direct effects on mitochondrial structure protection and GPX4 regulation.

Regarding the targets through which DAL inhibits ferroptosis, this study demonstrates that DAL upregulates GPX4, a key protective factor in the ferroptotic process. Ferroptosis regulatory networks involve multiple layers, including lipid peroxidation, iron metabolic dysregulation, and mitochondrial dysfunction. First, DAL suppresses I/R-induced ferroptosis by increasing GPX4 expression, which is critical for neutralizing lipid peroxides. Second, DAL restores the mitochondrial structure, possibly by regulating mitochondrial iron metabolism. Combined with the upregulation of HIF-1α, these effects suggest that DAL stabilizes the mitochondrial membrane potential and inhibits the iron-overload-induced opening of mitochondrial permeability transition pores, thereby blocking the execution phase of ferroptosis. This organelle-specific protective effect offers a new mechanistic perspective for DAL’s clinical efficacy. Transcriptomic and RT-qPCR analyses indicate that DAL may reduce lipid peroxidation substrates by inhibiting ACSL4-mediated arachidonic acid esterification [39]. As a multi-component herbal pair, DAL likely exerts anti-ferroptotic effects through multi-target mechanisms, such as regulating iron metabolism-related proteins to reduce intracellular iron accumulation, directly inhibiting lipid peroxidase activity to disrupt peroxidation cascades, or through synergistic interactions among its components. Initial experiments with astragaloside IV (AS-IV) and salidroside (SAL)—active components of Zhenqi Fuzheng Granules—showed suboptimal results, prompting us to use UHPLC-Q-Orbitrap HRMS combined with network pharmacology to identify DAL’s blood-enterable components associated with ferroptosis regulation, including Liquiritigenin, Rhamnocitrin, Sinapic acid, and 7,2’-dihydroxy-3’,4’-dimethoxyisoflavan. Subsequent molecular docking validation of binding affinity and interaction mode between these active components and targets provided structural-biological evidence supporting DAL’s renal protective mechanism via targeted regulation of ferroptosis-related pathways.

This study has several limitations. First, while HK-2 cells are widely used in renal tubular epithelial cell research, their relatively high resistance may lead to an underestimation of drug responses. Primary tubular cell experiments have shown greater sensitivity to hypoxia [36], highlighting the need for future validation in more physiologically relevant cell models. Second, the atypical dose–response relationship observed in DAL’s high-, medium-, and low-dose groups (18.2 g/kg, 9.1 g/kg, 4.55 g/kg) may be attributed to target saturation or metabolic rate limitation at high concentrations. This nonlinear response underscores the necessity of establishing a component–dose–effect relationship model via HPLC-MS/MS technology to clarify concentration-dependent effects. Finally, the dose–effect relationships of specific active components in DAL remain undefined, and key targets mediating its ferroptosis-inhibiting effects in I/R injury are unvalidated. Future studies should systematically dissect the component–target–pathway interactions using both animal and cell models to identify these potential targets, which are crucial for a comprehensive understanding of DAL’s mechanisms. While our findings provide preliminary evidence for DAL’s application in mitigating renal I/R injury, it is important to note that ferroptosis is only one pathological process in I/R injury. Whether DAL protects renal function through additional pathways—such as anti-inflammation, antioxidation, or anti-apoptosis—warrants further investigation.

## 4. Materials and Methods

### 4.1. Animals and Renal Ischemia/Reperfusion Model

MALE C57BL/6J mice (8 weeks old, 20 ± 3 g body weight) were purchased from Beijing Vital River Laboratory Animal Technology Co., Ltd., Beijing, China. The animals were housed under specific-pathogen-free conditions with a 12/12 h light/dark cycle, 50–60% relative humidity, and free access to food and water at 23 °C ± 2 °C. The mice were randomly divided into different groups with 5–6 mice in each group, anesthetized via an intraperitoneal injection of 50 mg/kg pentobarbital, placed on a 37 °C warming blanket, and depilated. A dorsal incision was made to expose the kidneys, and renal pedicles were clamped with curved forceps until the kidneys turned from bright red to dark purplish red, indicating successful ischemia. The model group underwent bilateral renal ischemia for 30 min, while the sham operation group only exposed the kidneys without pedicle clamping, and the remaining procedures were the same as those in the model group. After reperfusion (evidenced by restored renal redness), incisions were sutured, coated with liquid dressing, and mice received 0.4 mL/20 g saline via intraperitoneal injection for rehydration. Mice were first kept in a 37 °C incubator for warmth until awakening, then returned to the housing environment.

### 4.2. DAL and Monomer Interventions in I/R Mice

DAL consists of two botanical drugs, Astragali Radix and Ligustri Lucidi Fructus, which were purchased from Beijing Bencaofangyuan Pharmaceutical Co., Ltd., Beijing, China. For each herb, 30 g of the botanical material was mixed with tap water to form the mixture [40,41,42], which was then heated to 100 °C over high heat and maintained at a gentle boil over low heat for 20 min. To ensure the complete extraction of active components, this entire extraction process was repeated twice. The decoctions obtained from the two boiling cycles were combined and filtered through two layers of gauze to remove any remaining plant debris. The filtered decoction was transferred to an 80 °C water bath, where it was heated to promote evaporation and concentration (the volume of the final concentrated solution was calculated as 60 g/0.91 g·mL^−1^ = 66 mL). The concentrated decoction was stored at −20 °C to maintain stability and preserve its active ingredients. Before use, the decoction was thawed at room temperature and diluted to the required concentration for gavage administration. Interventions began on the day after modeling. The medium dose of DAL (DAL-M) was determined using the animal-to-human equivalent dose ratio. The low and high doses of DAL (DAL-L and DAL-H) were set at 1/2 and 2 times the medium concentration, respectively, with a gavage dosage of 0.2 mL/20 g. The calculation formula for DAL concentration based on the standard body weights of adult humans and mice is as follows:

The final concentration calculation of DAL-M (g/mL) = (Animal-to-human equivalent dose ratio) × (adult body weight/clinical dose)/ (gavage volume/mice body weight) = (9.1 mg/kg) × [60 kg/(30 g + 30 g)]/(0.2 mL/20 g) = 0.91 g/mL

For monomer groups, astragaloside IV (AS-IV) was administered at 30 mg/kg (AS-IV-L) or 60 mg/kg (AS-IV-H) [43], salidroside (SAL) at 30 mg/kg (SAL-L) or 60 mg/kg (SAL-H) [44], and the combination group (Com) received 60 mg/kg AS-IV + 60 mg/kg SAL, all via intraperitoneal injection. The positive drug group received 5 mg/kg Fer-1 (MedChemexpress, Monmouth Junction, NJ, USA). All doses were prepared using distilled water as the solvent, and the control group received an equal volume of distilled water. Interventions in each group lasted for 7 or 14 days, and the mice were euthanized after the last treatment. All animal procedures were approved by the Ethics Committee of Beijing University of Chinese Medicine (BUCM-2024091601–3274).

### 4.3. DAL Drug-Containing Serum Preparation

Twenty male SPF (specific-pathogen-free)-grade rats (200 ± 10 g) were purchased from Beijing Vital River Laboratory Animal Technology Co., Ltd., Beijing, China. The animals were housed under specific-pathogen-free conditions with controlled temperature (23 ± 2 °C), a 12/12 h light/dark cycle, 50–60% relative humidity, and ad libitum access to food and water. Following a 1-week acclimatization period, the rats were randomized into a control group and a DAL group (*n* = 10 per group). DAL was prepared as described in Section 4.2. The DAL group received daily oral gavage administration, while the control group received an equal volume of distilled water. The administration was conducted at a volume of 10 mL/kg per rat, and the calculation formula for the final concentration of DAL based on the standard body weights of adults and rats is as follows:

The final concentration calculation of DAL (g/mL) = (Animal-to-human equivalent dose ratio) × (adult body weight/clinical dose) /(gavage volume/rat body weight) = (6.3 mg/kg) × (60 kg/60 g)/(10 mL/kg) = 0.63 g/mL

Following 7 consecutive days of treatment, the rats were anesthetized with isoflurane, and blood samples were collected via abdominal aorta puncture. After allowing the blood to clot at room temperature for 1 h, serum containing DAL was isolated by centrifugation at 3000 rpm for 15 min at 4 °C. Serum samples were heat-inactivated at 57 °C for 30 min [45,46], filtered through a 0.22 μm membrane to remove bacteria, and stored at −80 °C for subsequent in vitro experiments.

### 4.4. Cell Culture, Treatment, and Viability Assay

HK-2 cells (human proximal renal tubular epithelial cell line) were obtained from Wuhan Shangen Biotechnology Co., Ltd., Wuhan, China. Cells were cultured in DMEM/F12 medium (Gibco, Billings, MT, USA) supplemented with 10% fetal bovine serum (FBS) and 1% penicillin–streptomycin (P/S), incubated in a 37 °C humidified incubator with 5% CO_2_. To prepare DAL-containing serum medium, fetal bovine serum (FBS) in normal DMEM/F12 medium was replaced with 5% or 10% DAL-containing serum. After routine passaging, cells were switched to sugar-free, serum-free DMEM/F12 medium (Procell, Wuhan, China) and transferred to a tri-gas incubator with 1% oxygen (balanced with nitrogen) for hypoxic incubation for 12 h. Subsequently, cells were returned to normal culture conditions with complete medium containing DAL-containing serum or 10 μM Fer-1 for reoxygenation, simulating in vivo I/R injury. For ferroptosis induction, cells were treated with complete medium containing 2 μM RSL3 (Shanghai Yuanye, Shanghai, China), followed by replacement with complete medium containing DAL-containing serum or 10 μM Fer-1 for different incubation durations. For live-cell mitochondrial fluorescence staining, cells were coincubated with complete medium containing either 2 μM RSL3 and DAL-containing serum or 10 μM Fer-1 for 3 h.

To measure cell viability, cells were seeded into 96-well plates at a density of 20,000 cells per well in 100 μL of culture medium. Hypoxic treatment or drug interventions were applied at specified time points, with the control group remaining untreated (no hypoxia or drug induction) and containing 6 technical replicates per group. 

Cell viability was assessed using the Cell Counting Kit-8 (CCK-8, Beyotime Biotechnology, Nantong, China) at 0, 3, 6, and 9 h post-treatment. Briefly, 10 μL of CCK-8 solution was added to each well, followed by incubation at 37 °C for 1 h. Absorbance at 450 nm was then recorded using a microplate reader (BioTek, Winooski, VT, USA).

### 4.5. Evaluation of Renal Function

Following euthanasia, blood was collected into non-heparinized plain centrifuge tubes, and kidneys and livers were harvested from the mice. Serum was isolated by centrifugation at 3000× *g* for 15 min at 4 °C. After rapid decapsulation, the kidneys were processed as follows: The intact left kidney was fixed in 4% paraformaldehyde for histological evaluation. The right kidney was divided into three parts: one part was snap-frozen in liquid nitrogen for biochemical analysis; another part was immersed in 2.5% glutaraldehyde dissolved in 0.1 M phosphate-buffered saline (PBS) for ultrastructural studies (observed via electron microscopy); and the third part was reserved for transcriptome sequencing. Intact liver tissues were fixed in 4% paraformaldehyde. Levels of serum creatinine (C011-2-1), blood urea nitrogen (C013-1-1), reduced glutathione (GSH, A006-2-1), malondialdehyde (MDA, A003-1-1), and tissue iron (A039-2-1) were measured using commercial kits (Nanjing Jiancheng Bioengineering Institute, Nanjing, China) according to the manufacturer’s instructions. Absorbance was recorded at the specified wavelengths using a microplate reader (BioTek, USA).

### 4.6. Histology

Kidney samples were fixed in 4% paraformaldehyde for 24 h at 4 °C, dehydrated through a graded ethanol series (70%, 80%, 95%, and 100%), cleared in xylene, and embedded in paraffin. Paraffin-embedded tissues were sectioned at 4 μm thickness using a rotary microtome (Leica, Wetzlar, Germany) and mounted on adhesive-coated glass slides. Hematoxylin and Eosin (H&E) Staining: Sections were deparaffinized, rehydrated, stained with hematoxylin for 5 min, differentiated in 1% acid alcohol, blued in ammonia water, and counterstained with eosin for 1 min. Periodic Acid–Schiff (PAS) Staining: Deparaffinized sections were oxidized in 0.5% periodic acid for 10 min, treated with Schiff reagent in the dark for 15 min, and counterstained with hematoxylin. Masson’s Trichrome Staining: Collagen fibers were stained blue using aniline blue, while muscle fibers and cytoplasm were stained red. Sirius Red Staining: Sections were incubated in 0.1% Sirius Red in saturated picric acid for 1 h. Renal injury severity was scored by two independent workers blinded to experimental groups according to a semi-quantitative scale: 0 (no injury), 1 (≤15% injury), 2 (16–30%), 3 (31–45%), 4 (46–60%), and 5 (>61%). Sirius Red- and Masson-stained sections were imaged at 200× magnification. Images were scanned using a super-resolution microtissue imaging system (Leica, Germany), photoprocessed using ImageScope x64 software (12.4), and positive areas were quantified using ImageJ software (fiji-win64). Five randomly selected fields per sample were analyzed. Fibrotic areas were quantified using ImageJ software by thresholding collagen-positive regions (blue for Masson; red for Sirius Red). Data were expressed as the percentage of fibrotic area relative to the total tissue area.

### 4.7. Immunofluorescence

Paraffin sections were dewaxed in xylene and hydrated through gradient ethanol (100%, 95%, 80%, 70%) followed by heat-mediated antigen retrieval using pH 8.0 EDTA at 100 °C for 20 min, then cooled to room temperature. Non-specific binding sites were blocked with 10% goat serum (Solarbio, Beijing, China) at 37 °C for 30 min. Sections were incubated overnight at 4 °C with primary antibodies against NGAL (Abcam, Cambridge, UK, ab216462, 1:100) and GPX4 (Abcam, ab125066, 1:100). After three PBS washes, corresponding Alexa Fluor 594/647-conjugated secondary antibodies (Cell Signaling Technology, Danvers, MA, USA) were applied for 1 h of incubation at 37 °C in darkness. Nuclei were counterstained with DAPI for 5 min before mounting with anti-bleaching reagent (Biorigin, Beijing, China). Images were acquired within 24 h using a confocal microscope (Olympus, Tokyo, Japan), and fluorescence intensity was quantified via ImageJ software.

### 4.8. Cryo-Electron Microscopy Sample Preparation and Imaging

Tissue samples were cut into small pieces and immersed in electron microscopy fixative (0.1 M PBS, pH 7.4) for 24 h of fixation at 4 °C. After primary fixation, samples were rinsed three times with 0.1 M PBS (15 min each), followed by post-fixation in 1% osmium tetroxide (dissolved in 0.1 M PBS, pH 7.4) at 20 °C for 2 h. Samples were rinsed again three times with PBS (15 min each). Dehydration was performed using a graded ethanol series (50%, 70%, 80%, 90%, 95%, and 100% ethanol and 100% acetone), with each step lasting 15 min. For infiltration, samples were incubated in graded mixtures of acetone and EPON 812 embedding medium (1:1 ratio for 2 h, 2:1 ratio overnight), followed by pure EPON 812 infiltration for 4 h. Samples were transferred to embedding molds, pre-polymerized at 37 °C overnight, and fully polymerized at 60 °C for 48 h. Ultrathin sections (60–80 nm) were prepared using a Leica UC7 ultramicrotome (Leica Microsystems, Singapore), stained with 2% aqueous uranyl acetate and lead citrate for 15 min each, and imaged using a Thermo Fisher Talos L120C transmission electron microscope (Thermo Fisher Scientific, Waltham, MA, USA) operated at a 120 kV acceleration voltage.

### 4.9. Western Blotting

Kidney and cell samples were lysed with RIPA buffer (Solarbio, China) containing protease inhibitors, followed by the addition of protein loading buffer. Proteins were separated via SDS-PAGE and transferred to PVDF membranes (Biorigin, China). Membranes were blocked with 5% non-fat milk in TBST and incubated overnight at 4 °C with primary antibodies against GPX4 (Abcam, ab125066, 1:1000), SLC7A11 (Abcam, ab307601, 1:1000), NGAL (R&D Systems, Minneapolis, MN, USA, AF1757, 1:100), and β-Actin (Abclonal, Woburn, MA, USA, AC026, 1:50,000). After three TBST washes, membranes were incubated with corresponding transmembrane antibodies: anti-rabbit IgG (Abcam, ab6721, 1:5000) for GPX4/SLC7A11/β-Actin and anti-goat IgG (Bioss, Woburn, MA, USA, bs-0294R-HRP, 1:1000) for NGAL. Protein bands were visualized using chemiluminescent ECL reagent (NCM Biotech, Shanghai, China) and detected with an electrochemical luminescence imaging system (Tanon-5200, Tanon Science & Techonlogy Co, Shanghai, China). Band intensities were quantified via ImageJ software.

### 4.10. Quantitative Real-Time PCR

Total renal RNA was isolated using an RNA extraction kit (Abclonal, Wuhan, China) following the manufacturer’s protocol for animal tissues. Extracted RNA was reverse-transcribed into cDNA using the Evo M-MLV reverse transcription system (Accurate Biology, Changsha, China). Reverse transcription–quantitative polymerase chain reaction (RT-qPCR) was performed with an SYBR Green Pro Taq HS premixed qPCR kit (Accurate Biology, Changsha, China) using the following primers: GPX4-F: CCCGATATGCTGAGTGTGGTTTA; GPX4-R: TTCTTGATTACTTCCTGGCTCCTG; PTGS2-F: CCAGCACTTCACCCATCAG; PTGS2-R: GATACACCTCTCCACCAATGAC; ACSL4-F: GCCATGGAAGCTGAAATACTGAAAG; ACSL4-R: GAAGGCATCTGTTACCAAACCAGTC; IL-6-F: CCGGAGAGGAGACTTCACAG; IL-6-R: CAGAATTGCCATTGCACAAC; IFN-γ-F: AGGTCAACAACCCACAGGTC; IFN-γ-R: ATCAGCAGCGACTCCTTTTC; TNF-α-F: ACTCCAGGCGGTGCCTATGT; TNF-α-R: GTGAGGGTCTGGGCCATAGAA; HiF-α-F: GGACGATGAACATCAAGTCAGCA; HiF-α-R: AGGAATGGGTTCACAAATCAGCA; α-SMA-F:CCCTGAAGAGCATCCGACAC; α-SMA-R: CCAGAGTCCAGCACAATACCA; LCN2(NGAL)-F: ATGTCACCTCCATCCTGGTC; LCN2(NGAL)-R: GCCACTTGCACATTGTAGCTC; KIM-1-F: CCAGGCGCTGTGGATTCTTA; KIM-1-R: TGACAAGCAGAAGATGGGCA; GAPDH-F: TGTGTCCGTCGTGGATCTGA; GAPDH-R: TTGCTGTTGAAGTCGCAGGAG

### 4.11. Transcriptome Sequencing

Total RNA was extracted from tissues using TRIzol^®^ Reagent following the manufacturer’s protocol, with quality assessment performed by Agilent 5300 Bioanalyzer and NanoDrop ND-2000 (Agilent, Santa Clara, CA, USA). Only high-quality RNA samples (OD260/280 = 1.8 ≥ 2.2, OD260/230 ≥ 2.0, RQN ≥ 6.5, 28S:18S ≥ 1.0, >1 μg) were used for library construction. Sequencing libraries were prepared at Shanghai Majorbio Bio-pharm Biotechnology Co., Ltd., Shanghai, China. using 1 μg total RNA via Illumina^®^ Stranded mRNA (Illumina, San Diego, CA, USA) Prep workflow, involving poly (A) selection, fragmentation, double-stranded cDNA synthesis with SuperScript kit (Thermo Fisher Scientific, Waltham, MA, USA), end repair, phosphorylation, adapter ligation, 300 bp size selection, and 15-cycle PCR amplification. Libraries were quantified by Qubit 4.0 and sequenced on either Illumina NovaSeq X Plus (PE150, NovaSeq Reagent Kit, Illumina, San Diego, CA, USA) or DNBSEQ-T7 (PE150, DNBSEQ-T7RS Reagent Kit v3.0, MGI Tech, Shenzhen, China). Raw reads were processed with fastp for quality control, aligned to the reference genome using HISAT2 in stranded mode, and assembled by StringTie. Gene expression was quantified by RSEM (TPM), with differential expression analysis performed using DESeq2 (|log2FC| ≥ 1, FDR < 0.05) or DEGseq (FDR < 0.001). The functional enrichment of GO terms and KEGG pathways was determined via Goatools (https://github.com/tanghaibao/GOatools (accessed on 28 March 2025)) and Python scipy (https://scipy.org/install/ (accessed on 28 March 2025)) with Bonferroni correction (*p* < 0.05). Alternative splicing events were identified using rMATS, focusing on exon inclusion/exclusion, alternative 5’/3’ splice sites, and intron retention events involving reference-matching or novel splice junctions. This version maintains technical details while complying with SCI formatting standards, using passive voice and academic tone appropriate for the Methods section. Key parameters, equipment models, and statistical thresholds are preserved. The transcriptome data have been deposited into NCBI’s Sequence Read Archive (SRA) database under the accession number PRJNA1249627.

### 4.12. Live-Cell Mitochondrial Fluorescence Staining

Live-cell mitochondrial staining was performed using a cell-permeable fluorescent dye specific to mitochondria (Genvivo, San Marino, CA, USA, PKMDR-1). Briefly, cells were cultured in glass-bottom dishes until they reached 70–80% confluence. Prior to staining, the culture medium was replaced with fresh pre-warmed medium containing the dye. Cells were incubated at 37 °C under 5% CO_2_ for 15 min to allow mitochondrial localization. Following incubation, cells were gently washed three times with phosphate-buffered saline (PBS) to remove excess dye. The nuclei were then labeled with Hoechst and washed three times with PBS. Live-cell imaging was immediately conducted using a confocal laser scanning microscope (Olympus, Japan). All experiments were repeated independently at least three times.

### 4.13. UHPLC-Q-Orbitrap-HRMS Analysis

DAL extract and drug-containing serum were centrifuged at 3000 rpm and 4 °C for 10 min, and 200 µL of supernatant was collected. The samples were further centrifuged at 12,000 rpm and 4 °C for 10 min, and the supernatant was stored at −20 °C until analysis. Chromatographic analysis was performed on a UHPLC-Q-Orbitrap HRMS system using an Agilent SB-C18 column (1.8 μm, 2.1 mm × 100 mm). The mobile phase consisted of 0.1% formic acid in water (A) and acetonitrile (B), with a linear gradient from 5% to 95% B over 0–9 min, a 9.1–10 min hold at 95% B, and re-equilibration. The flow rate was 0.35 mL/min, and the column temperature was maintained at 40 °C. MS analysis was performed on a Q Exactive Orbitrap mass spectrometer (Thermo Fisher Scientific, Waltham, MA, USA) in Full scan/dd-MS^2^ mode. Key parameters were set as follows: resolution, 70,000; AGC target, 1 × 10^6^; and collision energies, 10 V, 30 V, 60 V. Data were processed using Progenesis QI 3.0 software, and compounds were identified with the aid of TCM Pro 2.0 and theoretical databases. A Comprehensive Score above 55, Mass Error within ±6 ppm, and Isotope Similarity above 78 were set as the criteria for compound identification.

### 4.14. Network Pharmacological Analysis

Based on the UHPLC-Q-Orbitrap-HRMS results, network pharmacology analysis of DAL’s blood-enterable components was performed. The chemical structures of the identified prototype compounds in DAL were clarified using the PubChem database (https://pubchem.ncbi.nlm.nih.gov (accessed on 22 March 2025)), and potential targets of active components were predicted via the TargetPredictions database (http://www.swisstargetprediction.ch (accessed on 22 March 2025)), selecting targets with scores > 0. RIRI-related disease genes were retrieved from GeneCards (https://www.genecards.org (accessed on 24 March 2025)) and DisGeNET (https://www.disgenet.org (accessed on 24 March 2025)), with duplicate data removed. Drug active targets (scores > 0) and disease genes were imported into Venny 2.1.0 (https://bioinfogp.cnb.csic.es/tools/venny (accessed on 25 March 2025)) to identify intersecting genes, which were then uploaded to the STRING database (https://cn.string-db.org (accessed on 25 March 2025)) with the species restricted to Homo sapiens to construct a protein–protein interaction (PPI) network. Intersection genes were further analyzed via the DAVID database (https://david.ncifcrf.gov (accessed on 28 March 2025)) for GO functional and KEGG pathway enrichment, using “Homo sapiens” and “*p* < 0.05” as filtering criteria. Results were visualized through a bioinformatics platform (http://www.bioinformatics.com.cn (accessed on 29 March 2025)).

### 4.15. Molecular Docking

To determine the relationship between potential targets of DAL in treating RIRI and its active components, key active compounds of DAL and critical potential targets for RIRI treatment were identified via AutoDock Vina, integrating the results from “compound–target” networks, protein–protein interaction (PPI) networks, Gene Ontology (GO), and Kyoto Encyclopedia of Genes and Genomes (KEGG) enrichment analyses, including molecular docking.Inhibitors of potential targets were compared with screened active compounds. Structures of potential targets involved in RIRI treatment were sourced from the Protein Data Bank (PDB) and AlphaFold Protein Database. Ligands were preprocessed using AutoDock (4.2.6) Tools. Final conformations from docking simulations were verified and visualized using PyMOL (3.1) software.

### 4.16. Statistical Analysis

Statistical analyses were conducted using GraphPad Prism 8.0 software. Data normality was evaluated via Kolmogorov–Smirnov and Shapiro–Wilk tests. For data exhibiting normal distribution, values were presented as mean ± standard error of the mean (SEM). Group comparisons were performed using Student’s t-test or one-way analysis of variance (ANOVA) followed by Tukey’s post hoc test. Statistical significance was defined as *p* < 0.05.

## 5. Conclusions

This study reveals that DAL mitigates ischemia/reperfusion (I/R) injury by restoring intracellular GPX4 levels to resist ferroptosis, and screens potential pharmacodynamic components and drug targets, providing a theoretical basis for its clinical application. Future investigations are warranted to validate the experimental profiles of these components and their interactions with specific targets, thereby facilitating the modern development of this natural medicine.

## Data Availability

Data are contained within the article and Supplementary Material, and materials and data supporting this study’s findings are available from the first or corresponding authors upon reasonable request. The transcriptome data have been deposited into NCBI’s Sequence Read Archive (SRA) database under the accession number PRJNA1249627.

## Supplementary Materials

The following supporting information can be downloaded at: https://www.mdpi.com/article/10.3390/ph18060789/s1, Figure S1: The effects of long-term DAL use on I/R injury; Figure S2: DAL alleviates I/R injury by mediating ferroptosis; Figure S3: DAL alleviates I/R injury by mediating ferroptosis; Figure S4: The therapeutic effects of AS-IL and SAL on I/R; Figure S5: AS-IV and SAL have therapeutic effects on I/R and can protect mitochondrial morphology; Table S1: Identification of DAL; Table S2: Identification of blood-entering components of DAL.

## Abbreviations

IRI	Ischemia–reperfusion injury
I/R	Ischemia–reperfusion
RIRI	Renal ischemic reperfusion injury
AKI	Acute kidney injury
GPX4	Glutathione peroxidase 4
ROS	Reactive oxygen species
DAL	Drug pair of Astragali Radix–Ligustri Lucidi Fructus
MDA	Malondialdehyde
WB	Western blotting
AS-IV	Astragaloside IV
CCK-8	Cell Counting Kit-8
Fer-1	Ferrostatin-1
GSH	Glutathione
PPI	Protein–protein interaction
SAL	Salidroside
GO	Gene Ontology
GPX4	Glutathione peroxidase 4
OGD/R	Oxygen–glucose deprivation/reperfusion
TUNEL	TdT-mediated dUTP nick-end labeling
