# Supplementary material for: Drug Pair of Astragali Radix–Ligustri Lucidi Fructus Alleviates Acute Kidney Injury in Mice Induced by Ischemia–Reperfusion Through Inhibiting Ferroptosis
Source: Pharmaceuticals (Basel). 2025 May 25;18(6):789. doi: 10.3390/ph18060789 (PMC12195751; doi:10.3390/ph18060789)
Supplement: Supplementary file 1 [file pharmaceuticals-18-00789-s001.zip › pharmaceuticals-3627003-supplementary.pdf]

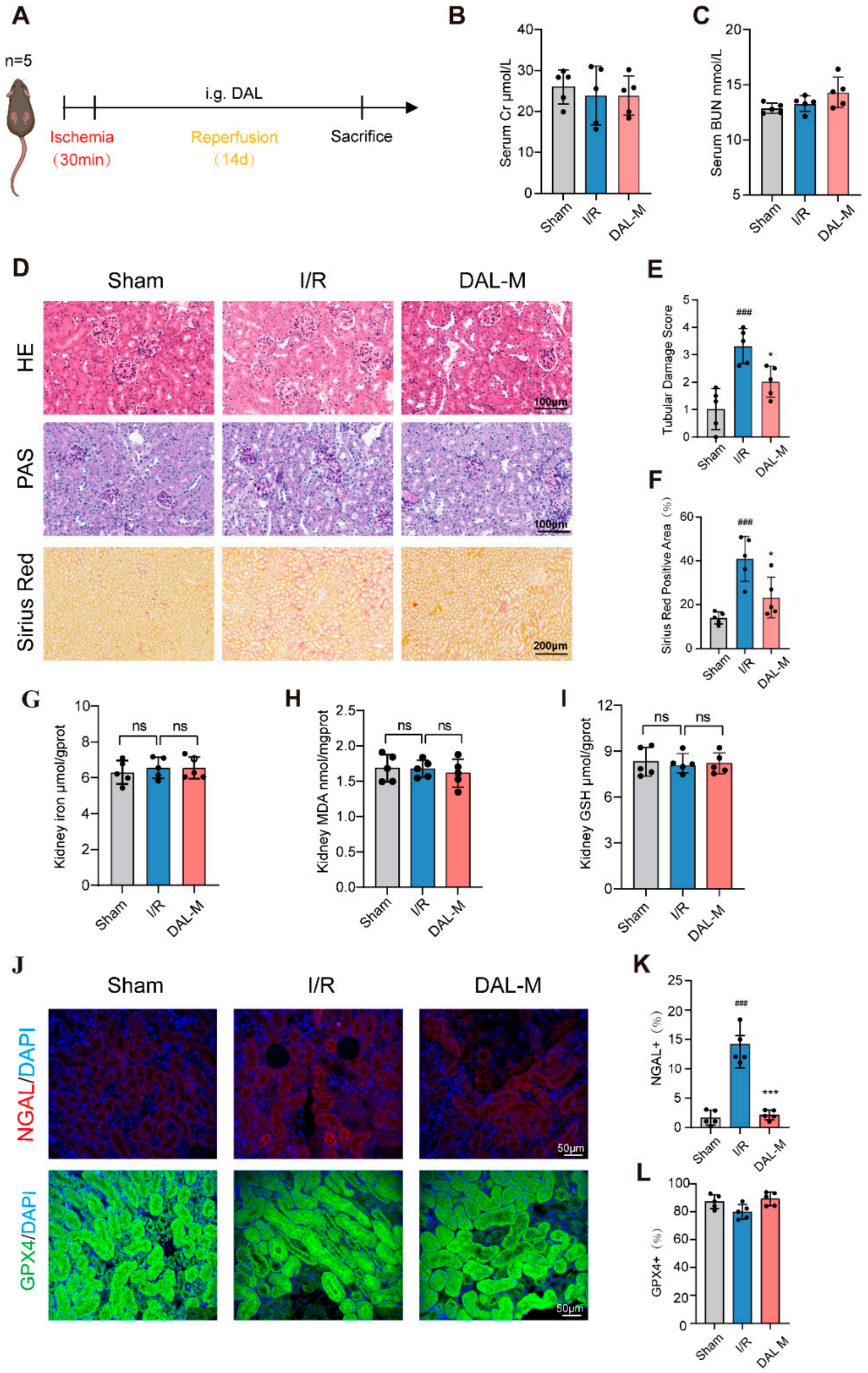

Figure S1. DAL alleviates post-I/R renal injury. (**A**) Schematics of the I/R experimental protocol (n=5);(**B-C**) Scr and BUN concentration;(**D**) Representative micrographs of H&E, PAS, and Sirius red staining. Scale bars, 100μm/200μm;(**E**) Quantification of tubular injury scores;(**F**) Quantification of Sirius red-positive area percentage.;(**G-I**) Levels of tissue iron, MDA, and GSH in mouse kidneys;(**J**) Representative immunofluorescence images of NGAL and GPX4. Scale bars, 50μm;(**K-L**) Quantification of NGAL- and GPX4-positive area percentages; I/R vs Sham:#p < 0.05, ##p < 0.01, ###p < 0.001;DAL vs I/R:*p < 0.05, **p < 0.01, ***p < 0.001;Data are presented as mean ± SEM.

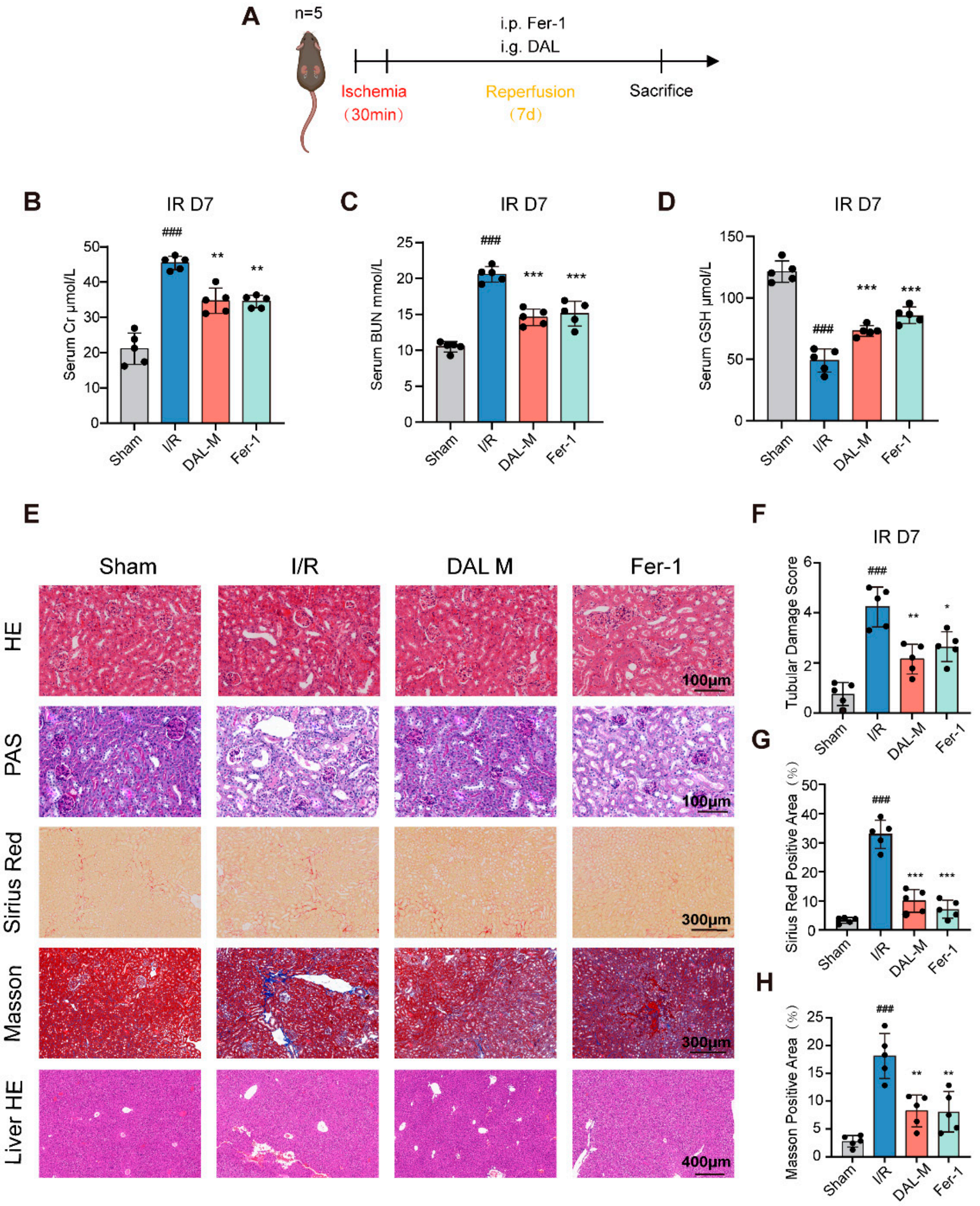

Figure S2. DAL alleviates I/R injury by mediating ferroptosis. (**A**) Schematics of the I/R experimental protocol (n=5);(**B-D**)Concentrations of Scr, BUN and GSH;(**E**) Representative micrographs of renal H&E, PAS, Sirius red, and Masson staining, as well as hepatic H&E staining. Scale bars, 100μm/300 μm/400μm;(**F**) Quantification of tubular injury scores;(**G**) Quantification of Sirius red-positive area percentage;(**H**)Quantification of Masson-positive area percentage;I/R vs Sham:#$p < 0.05$, ##$p < 0.01$, ###$p < 0.001$;DAL、Fer-1vs I/R:*$p < 0.05$, **$p < 0.01$, ***$p < 0.001$;Data are presented as mean ± SEM.

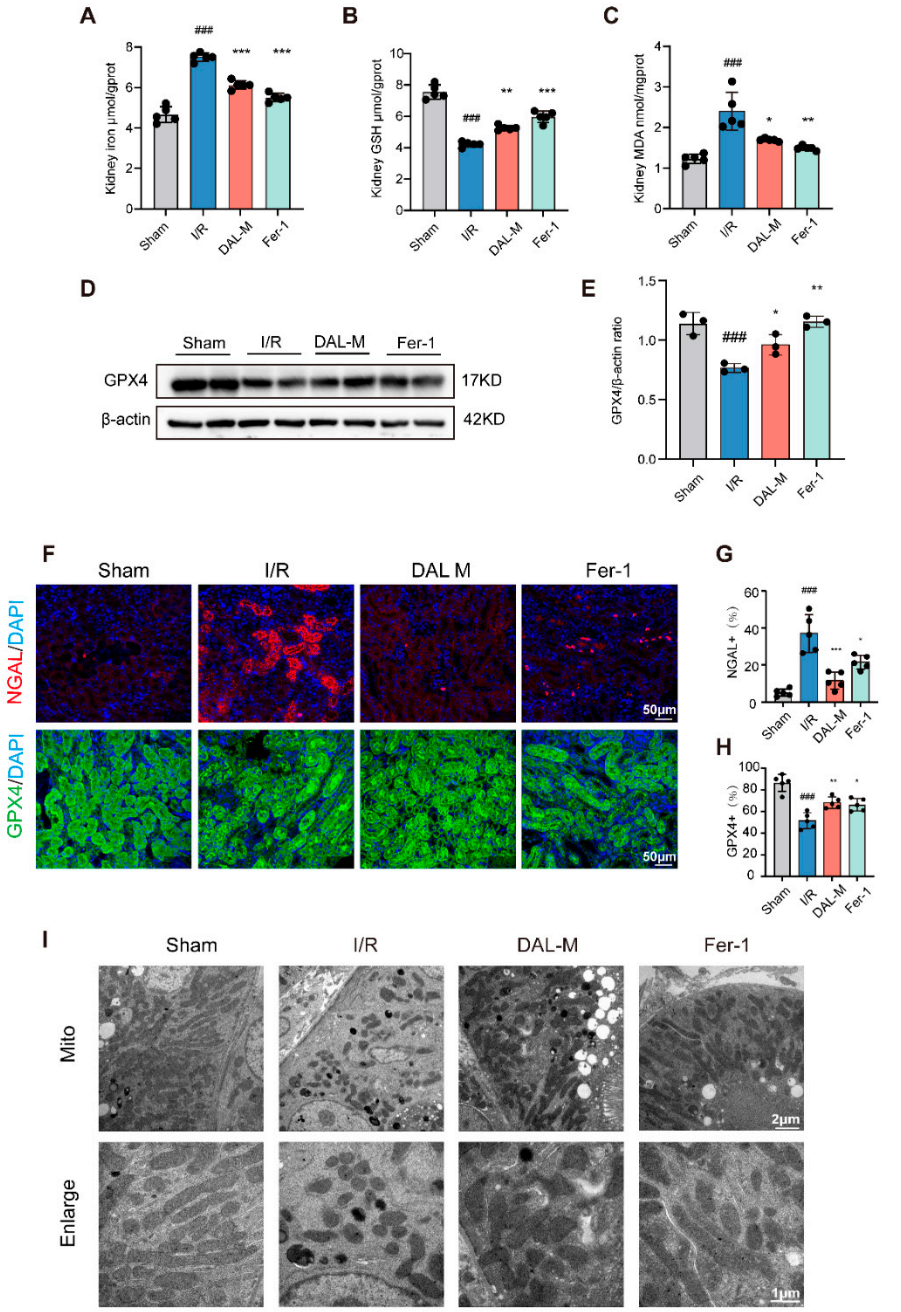

Figure S3. DAL alleviates I/R injury by mediating ferroptosis. (**A-C**)Levels of renal tissue iron, GSH, and MDA;(**D-E**) Western blot results showing GPX4 expression levels 7 days after I/R;(**F**)Representative immunofluorescence images of NGAL and GPX4;(**G-H**).Quantification of NGAL- and GPX4-positive area percentages. Scale bars, 50 μm;(**I**)Representative images of renal cortical mitochondria captured by transmission electron microscopy. Scale bars, 2 μm/1 μm;I/R vs Sham:#$p < 0.05$, ##$p < 0.01$, ###$p < 0.001$;DAL、Fer-1 vs I/R:*$p < 0.05$, **$p < 0.01$, ***$p < 0.001$;Data are presented as mean ± SEM.

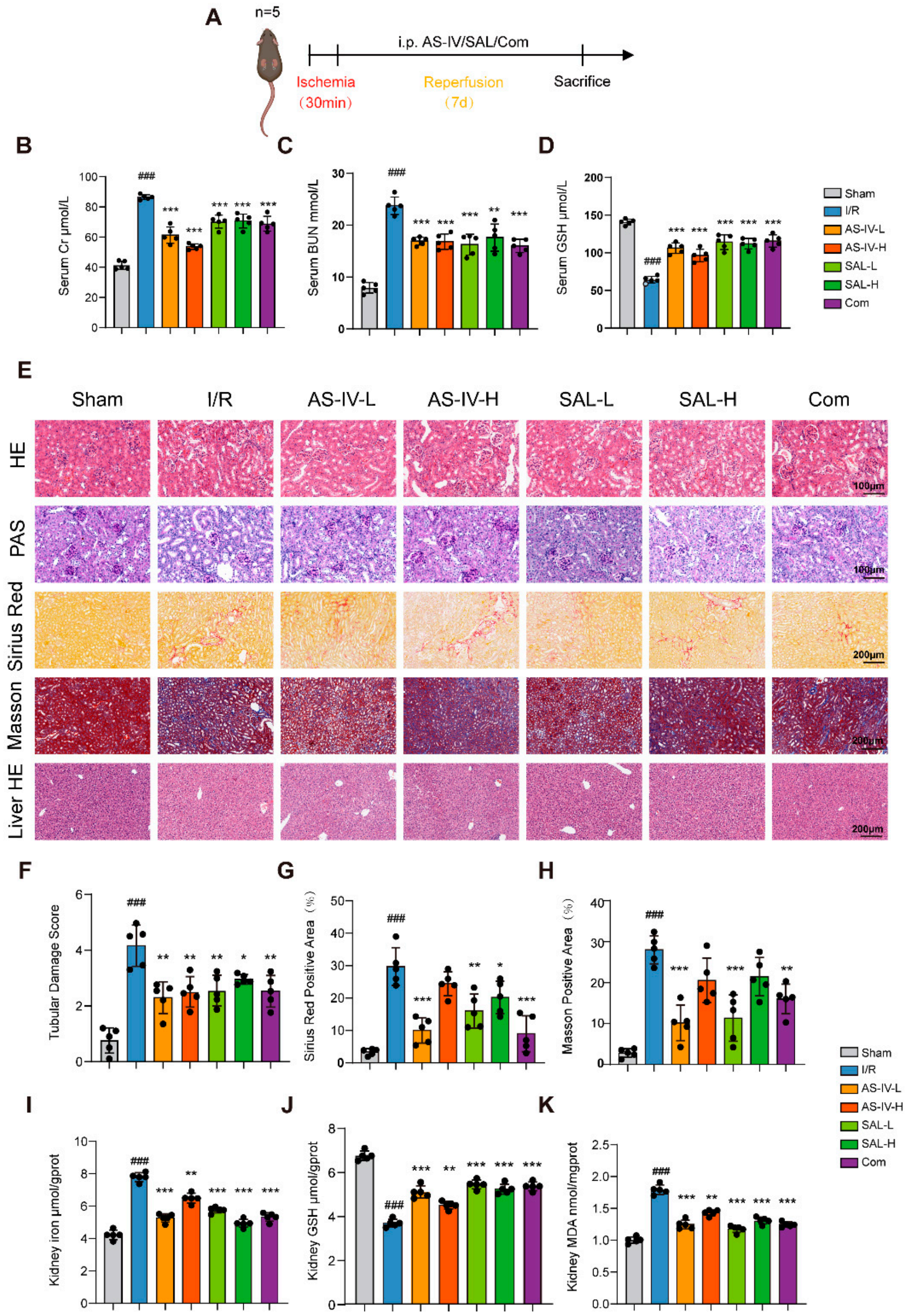

Figure S4. The therapeutic effects of AS-IL and SAL on I/R. (**A**)Schematics of the I/R experimental protocol (n=5) ;(**B-D**) Concentrations of Scr, BUN and GSH; (E) Representative micrographs of renal H&E, PAS, Sirius red, and Masson staining, as well as hepatic H&E staining. Scale bars, 100μm/200μm; (**F**) Quantification of tubular injury scores;(**G**) Quantification of Sirius red-positive area percentage;(**H**)Quantification of Masson-positive area percentage; (**I-K**)Levels of renal tissue iron, GSH, and MDA;I/R vs Sham:# $p < 0.05$, ## $p < 0.01$, ### $p < 0.001$，Chinese medicine monomers vs I/R:* $p < 0.05$, ** $p < 0.01$, *** $p < 0.001$.Data are presented as mean ± SEM.

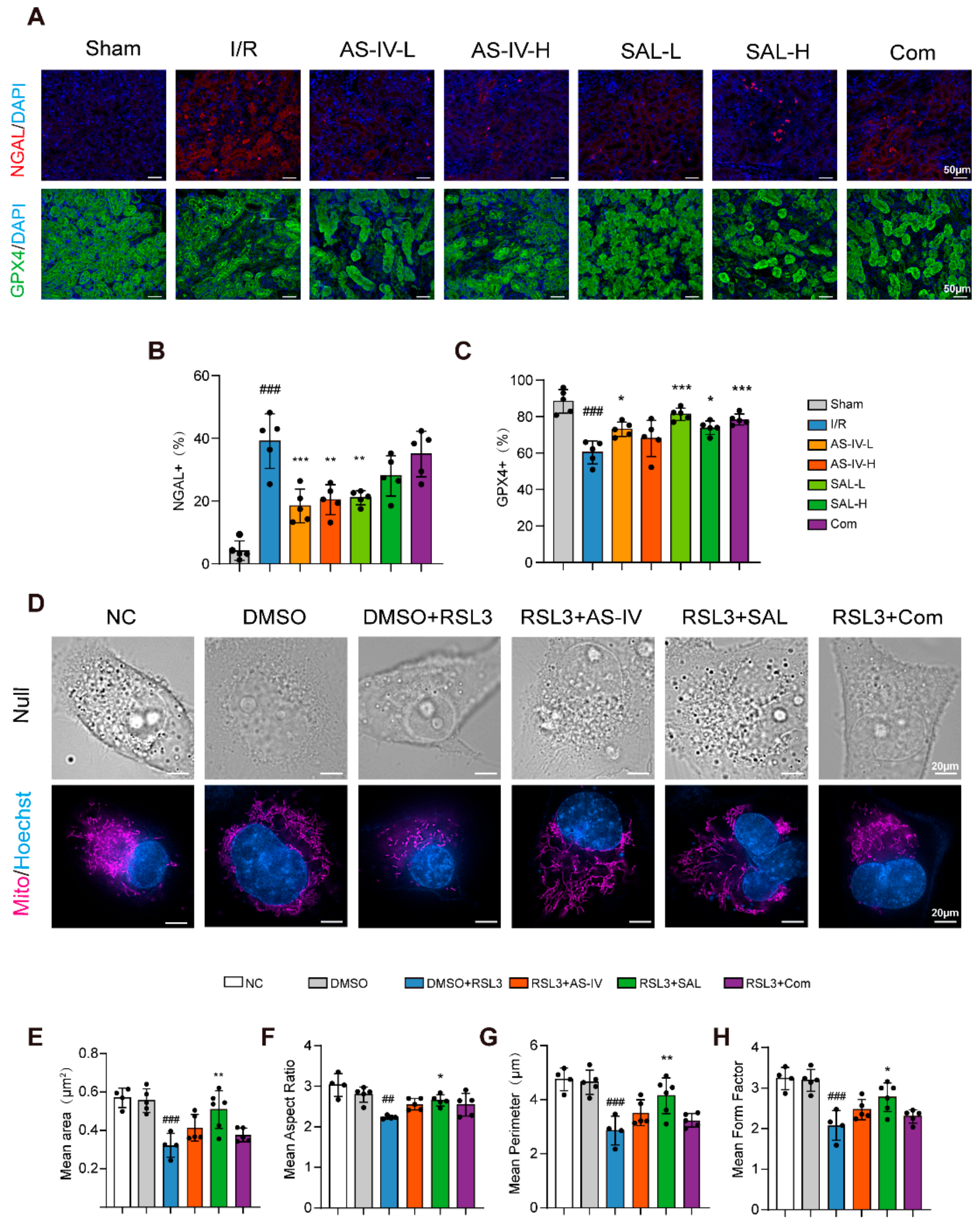

Figure S5. AS-IV and SAL have therapeutic effects on I/R and can protect mitochondrial morphology. (**A**) Representative immunofluorescence images of NGAL and GPX4;(**B-C**) Quantification of NGAL- and GPX4-positive area percentages. Scale bars, 50μm;(**D**)Representative images of mitochondrial fluorescence staining. Scale bars, 20μm;(**E-H**)Statistical analysis of mitochondrial morphological parameters;DMSO+RSL3 vs DMSO:#*p* < 0.05, ##*p* < 0.01, ###*p*< 0.001;RSL3+Chinese medicine monomers vs DMSO+RSL3 :**p* < 0.05, ***p* < 0.01, ****p* < 0.001;Data are presented as me mean ± SEM.

Table S1. Identification of DAL

NO.	Ion Mode	Retention time (min)	Discriminant	Experimental m/z	Theoretical m/z	Mass Error (ppm)	Fragment Score	Isotope Similarity	Comprehensive score	Formula	Name	Source	Identification by Reference substance
1	POS-NEG	11.60	M-H	623.1988	623.1981	1.00	85.50	99.32	93.63	C29H36O15	Acteoside/verbascoside/kusaginin	NMCC	Yes
2	POS	0.84	M+H-H2O, M+H	177.0978	177.0982	-2.28	71.80	94.40	65.88	C5H12N4O3	Canavanine	AR	
3	POS	0.91	M+H	116.0710	116.0706	3.15	65.60	94.66	64.00	C5H9NO2	Proline	AR	
4	POS	1.50	M+H	268.1037	268.1041	-1.43	66.10	98.02	65.63	C10H13N5O4	Adenine nucleoside	AR	
5	POS-NEG	1.54	M+H, 2M+H	284.0986	284.0990	-1.39	33.30	97.25	57.25	C10H13N5O5	Guanosine	AR	
6	POS-NEG	19.60	M+H	285.0751	285.0758	-2.49	84.10	95.30	93.38	C16H12O5	Biochanin a	NMCC	Yes
7	POS-NEG	5.42	M-H	451.1099	451.1093	1.27	51.90	98.31	62.25	C17H24O14	Nuezhenidic acid	LLF	
8	POS-NEG	11.00	M-H	417.1193	417.1191	0.44	82.00	92.94	92.25	C21H22O9	Liquiritin	NMCC	Yes
9	NEG	7.00	M-H	431.1560	431.1559	0.40	57.90	99.04	64.13	C19H28O11	Osmanthuside h	LLF	
10	POS-NEG	7.98	M-H	625.1422	625.1410	1.98	95.30	92.97	71.50	C27H30O17	Baimaside	LLF	
11	POS-NEG	14.60	M+H	257.0802	257.0809	-2.58	74.90	97.28	90.63	C15H12O4	Liquiritigenin	NMCC	Yes
12	POS-NEG	15.58	M+H, M+Na, M+K	285.0748	285.0758	-3.39	94.80	97.65	90.38	C16H12O5	Calycosin	AR	Yes
13	POS-NEG	8.59	M-H, M+FA-H	403.1248	403.1246	0.50	56.10	98.84	63.63	C17H24O11	Kingiside	LLF	
14	NEG	8.77	M-H	583.2044	583.2032	2.12	71.00	99.20	67.00	C27H36O14	Lucidumoside c	LLF	
15	POS	9.91	M+H	417.1170	417.1180	-2.37	95.40	92.39	90.00	C21H20O9	Daidzin	NMCC	Yes
16	POS-NEG	9.07	M+H-H2O, M+H	377.1449	377.1456	-1.70	30.70	93.00	55.50	C17H20N4O6	Riboflavin	AR	
17	NEG	19.59	M-H	513.1770	513.1766	0.71	75.20	94.18	88.25	C27H30O10	Baohuoside i	NMCC	Yes
18	POS-NEG	18.24	M+, M+H, M+Na	269.0800	269.0809	-3.02	92.00	97.90	88.00	C16H12O4	Formononetin	AR	Yes
19	POS-NEG	11.10	M+H, M+Na, 2M+H	447.1274	447.1286	-2.73	52.80	99.48	87.13	C22H22O10	Calycosin-7-o-β-d-glucoside	NMCC	Yes
20	NEG	10.05	M-H	609.1473	609.1461	2.00	78.40	91.44	66.88	C27H30O16	Neoisorutin	AR, LLF	
21	POS-NEG	10.09	M-H, M+FA-H	387.1300	387.1297	0.93	43.60	96.27	59.63	C16H22O8	Coniferin	LLF	

22	NEG	10.23	M-H	451.1615	451.1610	1.22	52.40	93.36	61.13	C22H28O10	4'-o-beta-d-glucosyl-5-o-methylvisamminol	AR	
23	NEG	17.87	M-H	255.0662	255.0663	-0.27	75.20	97.08	86.25	C15H12O4	Isoliquiritigenin	NMCC	Yes
24	POS-NEG	16.45	M+FA-H	867.2950	867.2928	2.49	92.70	94.01	85.38	C39H50O19	Epimedin c	NMCC	Yes
25	POS-NEG	10.88	M+FA-H	747.2373	747.2353	2.68	82.60	96.57	69.00	C31H42O18	Neonuezhenide	LLF	
26	POS-NEG	13.91	M-H	539.1772	539.1770	0.43	54.20	98.39	85.00	C25H32O13	Oleuropein	NMCC	Yes
27	POS-NEG	16.65	M+FA-H	721.2366	721.2349	2.26	85.80	96.45	83.75	C33H40O15	Icariin	NMCC	Yes
28	POS-NEG	12.26	M-H, M+FA-H	685.2353	685.2349	0.58	89.90	99.74	83.63	C31H42O17	Specnuezhenide	NMCC	Yes
29	NEG	10.95	M-H	609.1471	609.1461	1.61	91.30	98.56	83.50	C27H30O16	Rutin	NMCC	Yes
30	POS-NEG	12.46	M-H	431.0984	431.0984	0.22	93.80	95.14	83.50	C21H20O10	Sophoricoside	NMCC	Yes
31	POS-NEG	11.11	M-H	555.1721	555.1719	0.28	72.30	98.93	67.75	C25H32O14	10-hydroxyoleuropein	LLF	
32	POS-NEG	14.16	M+H, M+Na, M+K	431.1326	431.1337	-2.54	90.40	99.03	83.50	C22H22O9	Ononin	NMCC	Yes
33	POS-NEG	11.34	M+H-2H2O	341.1376	341.1383	-2.00	49.70	95.65	60.75	C20H24O7	(-)-olivir	LLF	
34	NEG	20.08	M-H	487.3436	487.3429	1.40	61.90	93.82	83.50	C30H48O5	Asiatic?acid	NMCC	Yes
35	POS-NEG	11.43	M+H	477.1383	477.1392	-1.83	33.10	97.20	57.00	C23H24O11	Astraisoflavan glucoside	AR	
36	POS-NEG	11.45	M-H, M+FA-H	731.2422	731.2404	2.40	72.30	98.55	67.00	C31H42O17	Nuezhenide	LLF	
37	POS-NEG	11.47	M-H	447.0938	447.0933	1.15	95.70	98.14	83.13	C21H20O11	Cynaroside/luteolin-7-o-glucoside/luteoloside	NMCC	Yes
38	POS	20.02	M+H-H2O, M+H	231.1375	231.1379	-1.88	58.50	97.66	83.00	C15H20O3	Atractylenolide iii	NMCC	Yes
39	POS-NEG	11.63	M-H, M+FA-H	477.1042	477.1039	0.73	63.60	93.69	64.13	C21H20O10	Cosmetin	LLF	
40	POS	11.89	M+H-2H2O, M+H-H2O, M+H	355.1015	355.1024	-2.51	66.20	96.56	65.00	C16H18O9	Heriguard	AR	
41	POS-NEG	16.93	M+H	271.0594	271.0601	-2.50	68.60	97.49	82.50	C15H10O5	Apigenin	LLF	Yes
42	NEG	12.26	M-H	623.1985	623.1981	0.54	79.60	98.05	69.25	C29H36O15	Acteoside	LLF	
43	POS-NEG	11.30	M+H	465.1020	465.1028	-1.70	97.90	96.90	81.25	C21H20O12	Isoquercitrin	NMCC	Yes

44	POS-NEG	12.27	M+H-2H2O, M+H-H2O, M+H	225.0750	225.0757	-3.40	57.70	97.88	63.00	C11H14O6	Kingiside aglucon	LLF	
45	POS	10.97	M+H	465.1018	465.1028	-2.11	73.10	91.90	80.75	C21H20O12	Hyperoside	NMCC	Yes
46	POS-NEG	12.44	M-H, M+FA-H	553.1569	553.1563	1.08	67.00	98.92	66.13	C25H30O14	Ligustrosidicacid	LLF	
47	POS	10.75	M+H-H2O, M+H	177.0544	177.0546	-1.24	76.20	97.97	79.88	C10H10O4	Ferulic acid	NMCC	Yes
48	NEG	19.58	M+FA-H	1075.5348	1075.5331	1.62	73.10	95.04	79.38	C51H82O21	Paris saponin vii	NMCC	Yes
49	NEG	12.64	M-H	541.1935	541.1926	1.55	55.70	92.06	61.50	C25H34O13	Lucidumoside b	LLF	
50	NEG	9.82	M+FA-H	727.2461	727.2455	0.82	65.30	88.13	78.63	C32H42O16	Pinoresinol diglucoside	NMCC	Yes
51	POS	11.08	M+H-2H2O, M+H-H2O, M+H	179.0698	179.0703	-2.58	51.60	97.35	78.38	C10H12O4	Cantharidin	NMCC	Yes
52	POS	3.43	M+H-H2O	109.0290	109.0284	5.58	85.70	98.55	77.38	C6H6O3	5-hydroxymethylfurfural	NMCC	Yes
53	NEG	14.50	M-H	299.0563	299.0561	0.70	51.50	97.24	62.00	C16H12O6	Rhamnocitrin	AR	
54	POS	19.05	M+H-2H2O, M+H-H2O, M+H	441.3718	441.3727	-2.06	57.40	94.10	77.38	C30H48O2	Roburic acid	NMCC	Yes
55	POS-NEG	14.67	M-H	539.1778	539.1770	1.49	46.10	97.29	60.38	C25H32O13	10-hydroxyligstroside	LLF	
56	NEG	14.71	M-H	287.0564	287.0561	1.08	41.70	96.65	59.25	C15H12O6	Eriodictyol	LLF	
57	POS-NEG	15.36	M+H	363.1428	363.1439	-2.87	49.10	95.63	60.38	C19H22O7	Ligustroside deglycosylation	LLF	
58	POS-NEG	15.37	M-H, M+FA-H	523.1819	523.1821	-0.25	48.30	98.68	61.63	C25H32O12	Methyl (4s,5e,6s) -5-ethylidene -4-[2-[2-(4-hydroxyphenyl)ethoxy] -2-oxoethyl] -6-[(2s,3r,4s,5s,6r) -3,4,5-trihydroxy -6-(hydroxymethyl)oxa	LLF	

											n -2-yl]oxy-4h-pyran-3-carboxylate		
59	POS-NEG	15.42	M-H, M+FA-H	1071.3565	1071.3562	0.30	67.80	99.65	66.75	C48H64O27	Oleonuezhenide	LLF	
60	POS	9.91	M+H, M+NH4, M+Na	804.2901	804.2921	-2.45	40.00	95.91	77.00	C35H46O20	Echinacoside	NMCC	Yes
61	NEG	15.44	M-H	685.2354	685.2349	0.75	74.00	94.38	66.88	C31H42O17	(8 e)-nüzhenide	LLF	
62	POS	15.45	M+H	463.1222	463.1235	-2.95	76.50	95.13	67.00	C22H22O11	Rhamnocitrin 3 -o-glucoside	AR	
63	NEG	17.65	M+FA-H	695.4027	695.4012	2.22	79.80	97.64	76.75	C36H58O10	Pedunculoside	NMCC	Yes
64	POS-NEG	15.64	M-H, M+FA-H	463.1616	463.1610	1.49	51.20	98.93	62.13	C23H28O10	Astraisoflavanin	AR	
65	POS	15.89	M+H	249.1478	249.1485	-2.80	53.10	96.88	61.75	C15H20O3	Santamarin	LLF	
66	POS-NEG	16.44	M+H	447.1279	447.1286	-1.66	37.00	97.67	58.13	C22H22O10	Calycosin 7 -o-glucoside	AR	
67	POS	25.03	M+H-H2O	439.3565	439.3570	-1.22	52.80	94.98	76.00	C30H48O3	Oleanolic acid	NMCC	Yes
68	NEG	12.51	M-H, M+FA-H	711.2519	711.2506	1.85	78.30	94.14	75.63	C33H44O17	(-)-syringaresnol-4-o-β-d-apiofuranosyl-(1→2)-β-d-glucopyranoside	NMCC	Yes
69	NEG	14.47	M-H	253.0505	253.0506	-0.27	63.20	95.28	75.50	C15H10O4	Daidzein	AR,LLF	Yes
70	POS	18.52	M+H-2H2O, M+H-H2O, M+H, M+Na	785.4663	785.4682	-2.40	31.80	95.37	75.13	C41H68O14	Astragaloside a/astragaloside iv	NMCC	Yes
71	NEG	17.31	M-H	945.5083	945.5064	1.97	83.10	98.37	69.75	C47H78O19	Astragaloside v	AR	
72	NEG	10.52	M-H	223.0605	223.0612	-3.08	95.70	97.93	74.63	C11H12O5	Sinapic acid	NMCC	Yes
73	NEG	17.54	M-H	553.1934	553.1926	1.33	77.70	97.36	68.38	C26H34O13	Ligustaloside a	LLF	
74	POS-NEG	12.31	M+H	579.1692	579.1709	-2.91	85.90	97.60	73.75	C27H30O14	Rhoifolin	NMCC	Yes
75	POS-NEG	17.72	M-H, M+FA-H	829.4613	829.4591	2.69	74.60	78.20	62.38	C41H68O14	Astragaloside iii	AR	
76	POS-NEG	17.76	M-H	377.1245	377.1242	0.99	45.90	95.67	60.13	C19H22O8	Oleuropein aglycone	LLF	
77	POS-NEG	17.49	M+H	301.0699	301.0707	-2.60	53.70	96.65	72.88	C16H12O6	Diosmetin	NMCC	Yes
78	NEG	19.27	M-H	503.3392	503.3378	2.80	67.00	92.68	72.13	C30H48O6	Madecassic acid	NMCC	Yes
79	POS-NEG	18.11	M+H-H2O, M+H	285.1690	285.1697	-2.52	52.90	93.86	72.00	C15H24O5	Dihydroartemisinin	NMCC	Yes

80	NEG	18.36	M-H, M+FA-H	871.4718	871.4697	2.45	71.80	88.78	64.38	C43H70O15	Isoastragaloside ii	AR	
81	NEG	16.65	M-H	271.0614	271.0612	0.97	35.20	95.84	71.38	C15H12O5	Naringenin	NMCC	Yes
82	POS	18.70	M+, M+H	303.1218	303.1227	-2.95	58.70	95.79	62.75	C17H18O5	7,2-dihydroxy -3',4'-dimethoxyisoflavan	AR	
83	NEG	18.79	M-H	839.4461	839.4434	3.11	50.10	84.65	57.75	C43H68O16	Cyclosiversioside b	AR	
84	POS	5.68	M+H	316.1537	316.1544	-2.10	81.00	95.50	71.25	C18H21NO4	Cephalotaxine	NMCC	Yes
85	POS	19.03	M+H-H2O, M+H	781.4714	781.4733	-2.37	55.80	93.10	61.50	C42H68O13	(3r,8s,9r,10r,13r,14s,17r) -3-hydroxy-4,4,9,13,14 -pentamethyl -17-[(e,2r)-6-methyl-7-[(2r,3r,4s,5s,6r) -3,4,5-trihydroxy -6-[[(2r,3r,4s,5s,6r) -3,4,5-trihydroxy -6-(hydroxymethyl)oxan -2-yl]oxymethyl]oxan-2-yl]oxyhept -5-en-2-yl]-1,2,3,7,8,10,12,15,16,17 -decahydr	AR	
86	POS-NEG	19.05	M-H, M+FA-H	941.5129	941.5115	1.42	95.00	99.31	73.13	C48H78O18	Soyasaponin i	AR	
87	NEG	18.95	M+FA-H	825.4664	825.4642	2.62	67.90	95.63	71.00	C42H68O13	Saikosaponin a	NMCC	Yes
88	POS	19.14	M+H-2H2O, M+H-H2O, M+H	455.3510	455.3520	-2.11	50.20	96.37	61.00	C30H48O4	19-α-hydroxy-3-acetyl-ursolic acid	LLF	
89	POS	19.14	M+H-2H2O, M+H-H2O, M+H	629.4034	629.4048	-2.15	72.60	97.23	66.88	C37H60O10	Astragaloside ii deglycosylation	AR	
90	POS	19.16	M+H-H2O	207.1375	207.1379	-2.00	37.00	97.73	58.13	C13H20O3	Vomifoliol	LLF	
91	POS-NEG	12.21	M+H	463.1222	463.1235	-2.76	73.60	92.23	69.13	C22H22O11	Tectoridin	NMCC	Yes

92	POS	0.84	M+H-H2O, M+H	175.1189	175.1190	-0.60	68.20	93.32	67.00	C6H14N4O2	Arginine	NMCC	Yes
93	POS	11.41	M+H-H2O, M+H	177.0543	177.0546	-1.78	48.00	97.16	65.38	C10H10O4	Isoferulic acid	NMCC	Yes
94	NEG	8.93	M+FA-H	433.1353	433.1352	0.40	30.90	95.71	64.75	C17H24O10	Verbenalin	NMCC	Yes
95	NEG	19.80	M-H	813.4662	813.4642	2.47	92.00	93.72	70.75	C42H70O15	Astramembranoside a	AR	
96	POS-NEG	19.87	M+H-2H2O, M+H-H2O, M+Na	293.2104	293.2111	-2.53	31.20	96.79	56.25	C18H30O4	6-methylgingediol	LLF	
97	POS-NEG	15.42	M+H	287.0541	287.0550	-3.24	40.90	96.83	63.88	C15H10O6	Luteolin	LLF	Yes
98	POS	8.53	M+H-H2O	181.0493	181.0495	-1.27	36.20	96.89	63.25	C9H10O5	Syringic acid	NMCC	Yes
99	NEG	20.30	M-H	489.3591	489.3585	1.15	51.80	91.12	60.38	C30H50O5	Cycloastragenol	AR	
100	POS-NEG	20.63	M-H, M+FA-H	487.3435	487.3429	1.19	54.80	98.53	63.00	C30H48O5	Tormentic acid	LLF	
101	NEG	22.25	M-H	295.2279	295.2278	0.16	68.40	97.63	66.50	C18H32O3	(s)-coriolic acid	AR	
102	POS	22.53	M+H-H2O, M+H	455.3513	455.3520	-1.37	62.50	97.73	64.63	C30H48O4	2alpha,3beta -dihydroxyolean -12-en-28-oic acid	LLF	
103	NEG	24.57	M-H	513.3585	513.3585	-0.05	73.70	97.48	67.75	C32H50O5	3-acetylpomolic acid	LLF	
104	NEG	8.14	M-H	403.1252	403.1246	1.49	49.40	95.02	62.88	C17H24O11	Hastatoside	NMCC	Yes
105	NEG	6.15	M-H, M+FA-H	345.1192	3451.1928	0.29	26.00	96.61	62.63	C14H20O7	Salidroside	LLF	Yes

（AR：Astragali Radix；LLF：Ligustri Lucidi Fructus；NMCC：Non-medicinal characteristic compounds）

Table S2. Identification of blood-entering components of DAL

No.	Name of Prototype	Transformations	Ion Mode	Retention time (min)	Experimental m/z	Peak Area	Source
DAL001	19-α-hydroxy-3-acetyl-ursolic acid	-COO	POS	21.63	429.3720	49485.68	LLR
DAL002	4'-o-beta-d-glucosyl-5-o-methylvisamminol	-H2	NEG	5.37	495.1516	1675.907272	AR
DAL003	7,2-dihydroxy -3',4'-dimethoxyisoflavan	-H2	POS	15.28	301.1067	43033.60	AR
DAL004	Acteoside	+H2	NEG	9.83	671.2213	6369.24147	LLF
DAL005	Astragaloside iii		NEG	18.52	829.4616	40511.97	AR
DAL006	Astraisoflavanin	-H2+O2	NEG	11.71	493.1363	19476.10	AR
	Astraisoflavanin	+C6H8O6	NEG	12.78	639.1944	271338.8187	AR
DAL007	Biochanin a	+C6H8O6	NEG	12.47	459.0941	1567810.037	NMCC
DAL008	Calycosin 7-o-glucoside	+C6H8O6	NEG	9.29	621.1476	17555.6869	AR
	Calycosin 7-o-glucoside	+H2O	NEG	12.07	463.1258	21880.01	AR
	Calycosin 7-o-glucoside	+O-H2	POS	12.47	461.1075	628103.17	AR
DAL009	Canavanine		POS	0.84	177.0980	9374.77	AR
DAL010	Cephalotaxine	+O-CO	POS	4.30	304.1540	8355.45	NMCC
DAL011	Coniferin	+O-H2	NEG	6.76	355.1040	6371.65	LLF
	Coniferin	+O3	NEG	7.39	389.1097	6815.82	LLF
	Coniferin	+O	NEG	7.64	357.1199	2995.72	LLF
	Coniferin	-H2	NEG	13.78	339.1089	10719.61	LLF
DAL012	Cosmetin		NEG	10.81	431.0990	4244.762043	LLF
	Cosmetin	+CH2	NEG	14.52	445.1148	49547.13	LLF
	Cosmetin	+CH2	NEG	14.75	491.1205	12080.15	LLF
DAL013	Cynaroside		NEG	12.30	447.0941	11130.17372	NMCC
DAL014	Daidzein	+C6H8O6	NEG	11.97	429.0832	4328.147432	AR,LLF
DAL015	Formononetin	+O	NEG	12.47	283.0607	1439.30	AR
	Formononetin	+C6H8O6	NEG	12.97	489.1047	4565.356969	AR
	Formononetin	+C6H8O6	POS	14.17	445.1127	64823.53	AR
	Formononetin	+C6H8O6	NEG	14.18	443.0992	147217.83	AR

	Formononetin		NEG	18.23	267.0664	6326.958265	AR
DAL016	Isoferulic acid	+H2O-CO	POS	7.27	185.0808	15722.74	NMCC
	Isoferulic acid	-CO	POS	15.41	167.0702	10605.10	NMCC
DAL017	Kingiside	-H2+O2	NEG	6.36	433.0992	6620.56	LLF
	Kingiside		NEG	8.61	403.1252	2690.291512	LLF
DAL018	Kingiside aglucon	+H2O	NEG	6.04	259.0825	2155.04	LLF
	Kingiside aglucon	+O-CO	NEG	7.75	229.0713	6350.64	LLF
	Kingiside aglucon		NEG	10.56	241.0715	7005.716126	LLF
DAL019	Liquiritigenin	+H2	NEG	11.19	303.0878	3457.48589	NMCC
	Liquiritigenin	+CO	POS	12.47	285.0754	78864.88	NMCC
DAL020	Liquiritin	+CH2	NEG	15.41	477.1409	616161.375	NMCC
DAL021	Lucidumoside c	-H2O	NEG	10.87	565.1936	4702.69	LLF
DAL022	Neoisorutin		NEG	10.49	609.1479	4513.383904	AR, LLF
DAL023	Nuezhenidic acid		NEG	5.33	451.1100	1440.763306	LLF
DAL024	Ononin	+O2	NEG	11.39	461.1101	5634.16	NMCC
	Ononin	+H2	NEG	17.28	431.1352	24659.93	NMCC
DAL025	Osmanthuside h	-H2	NEG	4.67	475.1464	8384.282343	LLF
DAL026	Rhamnocitrin	+C6H8O6	NEG	10.51	475.0889	4798.77055	AR
	Rhamnocitrin	+CH2	NEG	19.62	313.0724	2334.33	AR
DAL027	Rhoifolin	+O3	NEG	9.66	625.1423	14683.83	NMCC
DAL028	Riboflavin	+O-CO	NEG	4.75	363.1304	18632.94	AR
DAL029	Sinapic acid	+H2O-CO	NEG	6.29	213.0759	8967.61	NMCC
	Sinapic acid	+SO3	NEG	7.23	303.0184	4904.13	NMCC
	Sinapic acid	+C6H8O6	NEG	7.50	399.0938	2941.225245	NMCC
	Sinapic acid	+O	NEG	7.63	239.0559	48358.48	NMCC
DAL030	Sophoricoside	+O	NEG	10.08	447.0942	8166.98	NMCC
	Sophoricoside		NEG	12.87	477.1046	5914.586548	NMCC
DAL031	Tectoridin	+O-CO	NEG	11.34	449.1092	11323.01	NMCC
	Tectoridin		NEG	12.97	461.1100	35140.46808	NMCC
	Tectoridin	+CH2	NEG	15.27	475.1252	746928.10	NMCC

	Tectoridin	+CH2	POS	15.28	477.1389	218894.38	NMCC
	Verbenalin	+H2O-CO	NEG	8.98	377.1460	7981.68	NMCC
DAL032	Verbenalin	+CH2	NEG	13.32	401.1459	2803.97	NMCC
	Verbenalin	-H2O	NEG	13.46	369.1196	8263.20	NMCC

（AR：Astragali Radix；LLF：Ligustri Lucidi Fructus；NMCC：Non-medicinal characteristic compounds）
